# Mechanical Property Prediction of Industrial Low-Carbon Hot-Rolled Steels Using Artificial Neural Networks

**DOI:** 10.3390/ma18132966

**Published:** 2025-06-23

**Authors:** Saurabh Tiwari, Hyoju Ahn, Maddika H. Reddy, Nokeun Park, Nagireddy Gari S. Reddy

**Affiliations:** 1 School of Materials Science and Engineering, Yeungnam University, Gyeongsan 38541, Republic of Korea; saurabht@yu.ac.kr (S.T.); ahnalysis@yu.ac.kr (H.A.); 2 Department of Mechanical Engineering, CVR College of Engineering, Hyderabad 500029, Telangana, India; harinathareddy.maddika@cvr.ac.in; 3 Institute of Materials Technology, Yeungnam University, Gyeongsan 38541, Republic of Korea; 4 Department of New Materials Engineering, Engineering Research Institute, Gyeongsang National University, Jinju 52828, Republic of Korea

**Keywords:** artificial neural networks, hot-rolled steel strip, mechanical property prediction, finish rolling temperature, coil target temperature, yield strength, ultimate tensile strength, elongation, low-carbon steel

## Abstract

This study investigated the application of neural network techniques to predict the mechanical properties of low-carbon hot-rolled steel strips using industrial data. A feedforward neural network (FFNN) model was developed to predict the yield strength (YS), ultimate tensile strength (UTS), and elongation (%EL) based on the chemical composition and processing parameters. For the low-carbon hot-rolled steel strip (C: 0.02–0.06%, Mn: 0.17–0.38%), 435 datasets were utilized with 17 input parameters, including 15 composition elements, finish rolling temperature (FRT), and coil target temperature (CTT). The model was trained using 335 datasets and tested using 100 randomly selected datasets. The optimum network architecture consisted of two hidden layers with 34 neurons each, achieving a mean squared error of 0.014 after 200,000 iterations. The model predictions showed excellent agreement with the actual values, with mean percentage errors of 4.44%, 3.54%, and 4.84% for the YS, UTS, and %EL, respectively. The study further examined the influence of FRT and CTT on mechanical properties, demonstrating that FRT has more complex effects on mechanical properties than CTT. The model successfully predicted property variations with different processing parameters, thereby providing a valuable tool for alloy design and process optimization in steel manufacturing.

## 1. Introduction

The mechanical properties of steel products are significantly influenced by their chemical composition and processing parameters. Establishing predictive relationships between the composition, processing conditions, and final properties is crucial for optimizing steel manufacturing processes. Traditional empirical approaches and physical metallurgy principles have been useful but often fail to capture complex, non-linear relationships in modern steelmaking operations [1,2]. In recent years, machine learning techniques, particularly artificial neural networks (ANNs), have emerged as powerful tools for modeling complex relationships in materials science [3,4,5,6]. Neural networks can effectively handle multiple input parameters and capture nonlinear interactions without requiring explicit mathematical relationships between inputs and outputs [7,8]. This capability makes them particularly suitable for predicting the mechanical properties of steels in which multiple compositional elements and processing parameters interact in complex ways [9,10]. Hot-rolled steel strips are widely used in various applications, including automotive components, construction materials, and consumer goods. Hot-rolled steel strips are widely used in various industries because of their versatility and mechanical properties. These strips play a crucial role in the automotive manufacturing, chemical, and home appliance industries, and their surface quality significantly affects the final product [11,12]. The construction industry extensively utilizes hot-rolled steel sections for structural elements and complex steel nodes in bridges and truss structures [13,14]. The mechanical properties of hot-rolled steel strips, such as yield strength (YS), ultimate tensile strength (UTS), and elongation (EL), are critical for their performance in service. These properties are influenced by numerous factors, including chemical composition, finish rolling temperature (FRT), coil target temperature (CTT), and other processing parameters [15,16,17,18].

The application of neural networks in the field of materials science, particularly in predicting the properties of steel, has gained significant traction over the past few decades. The ability of these computational models to analyze the complex relationships between various parameters has opened new avenues for research and development in metallurgy. The pioneering work of Singh and Bhadeshia [19] laid the groundwork for utilizing neural networks to model bainite plate thickness in low-alloy steels. Their study demonstrated that neural networks can effectively capture the nonlinear relationships inherent in the microstructural evolution of steel, which is critical for understanding its mechanical properties. The authors employed a feedforward neural network architecture, which was trained on experimental data, to predict the bainite thickness based on various input parameters such as alloy composition and cooling rates. This early application highlights the potential of neural networks as robust predictive tools in materials science. Chang and Bhadeshia further explored these relationships in their research [20]. Recently, Wang et al. [21] developed models to predict the mechanical properties of hot-rolled steel plates. The NGBoost algorithm was used to predict the mechanical properties of hot-rolled strip steel, including tensile strength, yield strength, and elongation, while employing ANOVA to identify the significant factors affecting mechanical performance, design enhancement, and safety. In contrast, Wu et al. [22] did not focus on interpretable machine learning to predict the mechanical properties of hot-rolled strip steel. Instead, it utilizes model-agnostic meta-learning to enhance prediction accuracy and adaptability with limited data, effectively addressing small-sample problems. Fang et al. [23] provided a comprehensive review of machine learning applications in steel research. However, most studies have focused on a limited number of input parameters or specific steel grades without comprehensively examining the influence of processing temperature on the composition.

In this study, a data-driven artificial intelligence model was developed to predict the key mechanical properties of low-carbon hot-rolled steel strips: YS, UTS, and EL (%). Unlike previous approaches that focused on a limited set of inputs, this model integrates 15 alloying elements along with two critical processing parameters, FRT and CTT, allowing for the modeling of complex nonlinear interactions that govern mechanical behavior. This comprehensive input selection enables a more realistic and actionable understanding of how composition and processing jointly influence steel performance.

To enhance usability and industrial relevance, a user-friendly graphical user interface (GUI) was developed. This tool allows users to input parameters, predict mechanical properties, and perform sensitivity analyses without requiring programming skills or prior knowledge of artificial neural networks. This accessibility makes the model a practical tool for researchers, engineers, and technicians involved in steel design and process optimization. Together, the model’s depth, interpretability, and ease of use represent a novel contribution to the field of computational materials engineering and support the broader adoption of AI tools in smart manufacturing.

## 2. Materials and Methods

### 2.1. Data Collection and Preprocessing

The hot-rolled steel strip dataset used in this study was obtained through an industrial–academic collaboration with Tata Steel’s Jamshedpur facility, one of the largest integrated steel plants in India. Data were collected from in-plant trials and routine production runs encompassing a diverse range of alloy compositions and thermomechanical processing parameters. This dataset reflects real-world manufacturing conditions and provides a robust foundation for analyzing the relationship between the processing, composition, and mechanical properties. A total of 435 datasets (Supplementary Material: Appendix: Hot-rolled steel) were collected, including chemical composition data (15 elements), finish rolling temperature (FRT), coil target temperature (CTT), and the corresponding mechanical properties: yield strength (YS), ultimate tensile strength (UTS), and elongation percentage (EL). Chemical composition analysis included aluminum (Al), soluble aluminum (ALS), carbon (C), chromium (Cr), copper (Cu), manganese (Mn), molybdenum (Mo), nitrogen (N), niobium (Nb), nickel (Ni), phosphorus (P), sulfur (S), silicon (Si), titanium (Ti), and vanadium (V). The compositions and processing parameters used in this study are listed in Table 1. The tensile test data used in this study were collected at the Jamshedpur facility of Tata Steel. Based on standard practices, an Instron 5500 Series Universal Testing Machine, widely used in the industry and compliant with ASTM E8/IS 1608, was employed for mechanical testing.

**Figure 1:**
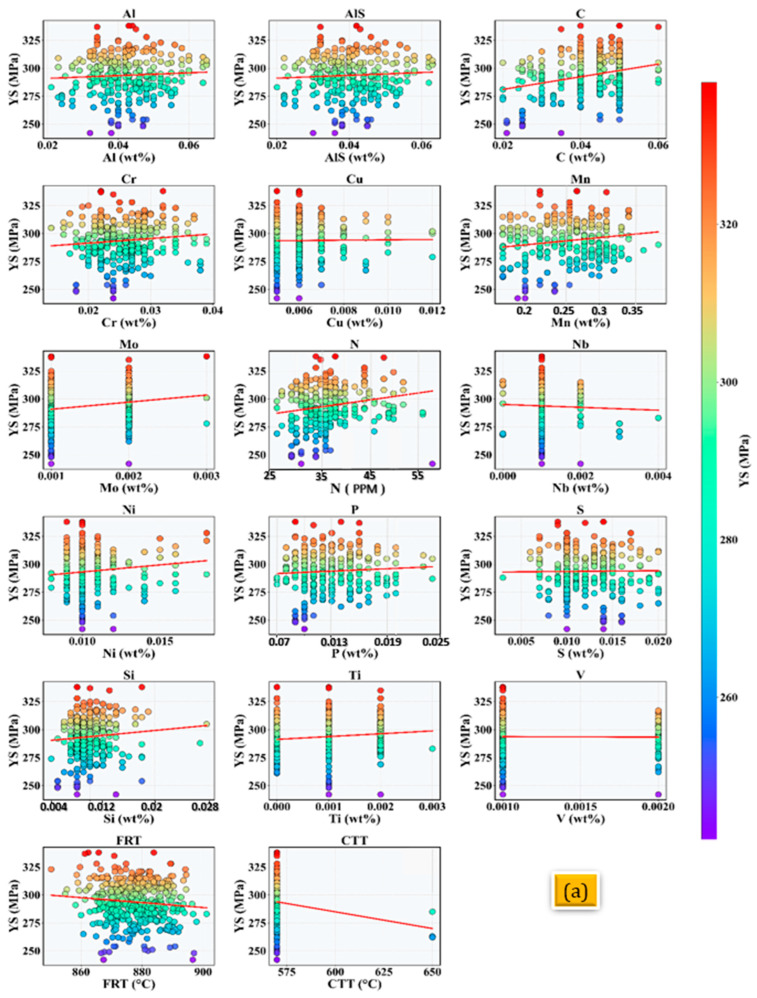

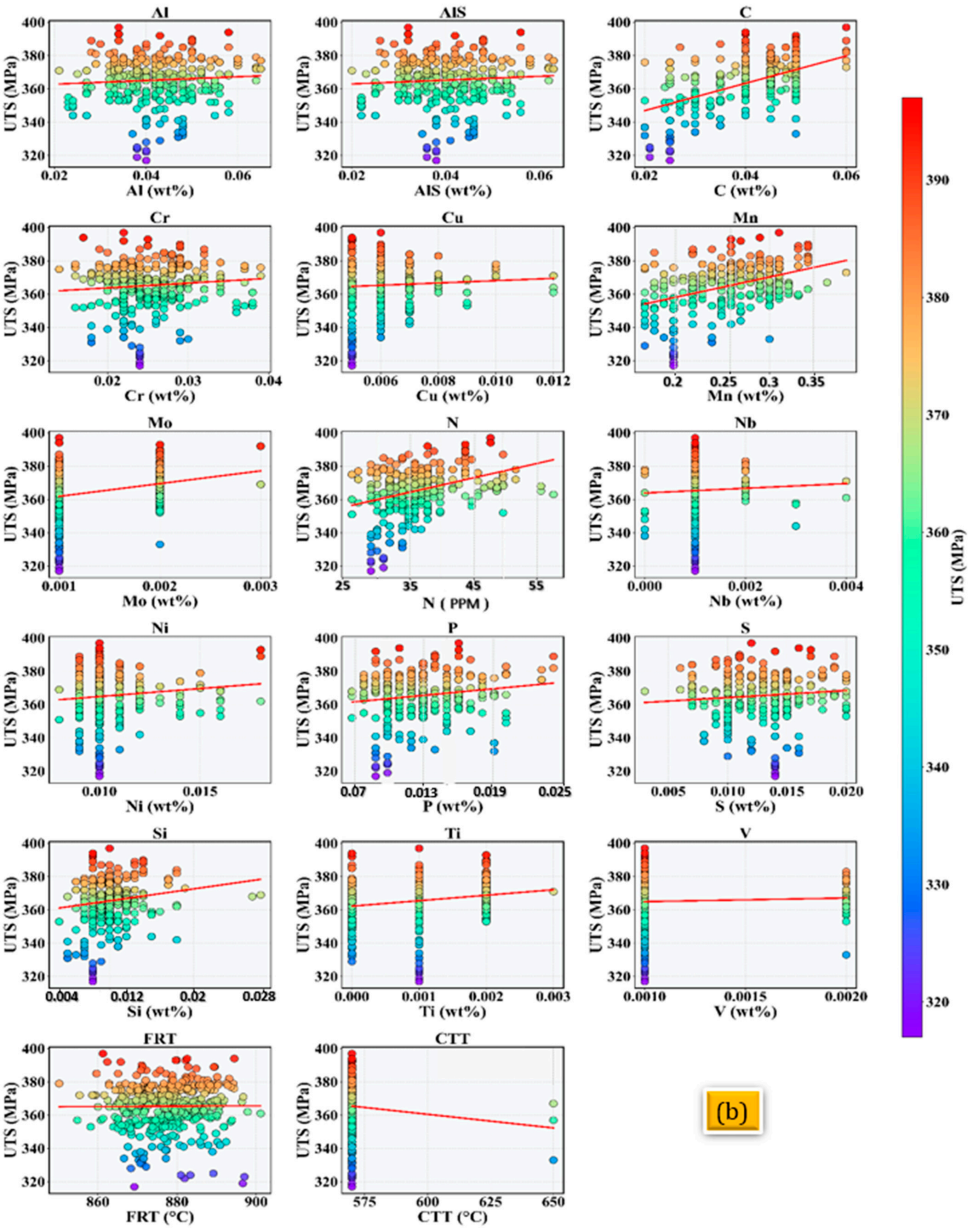

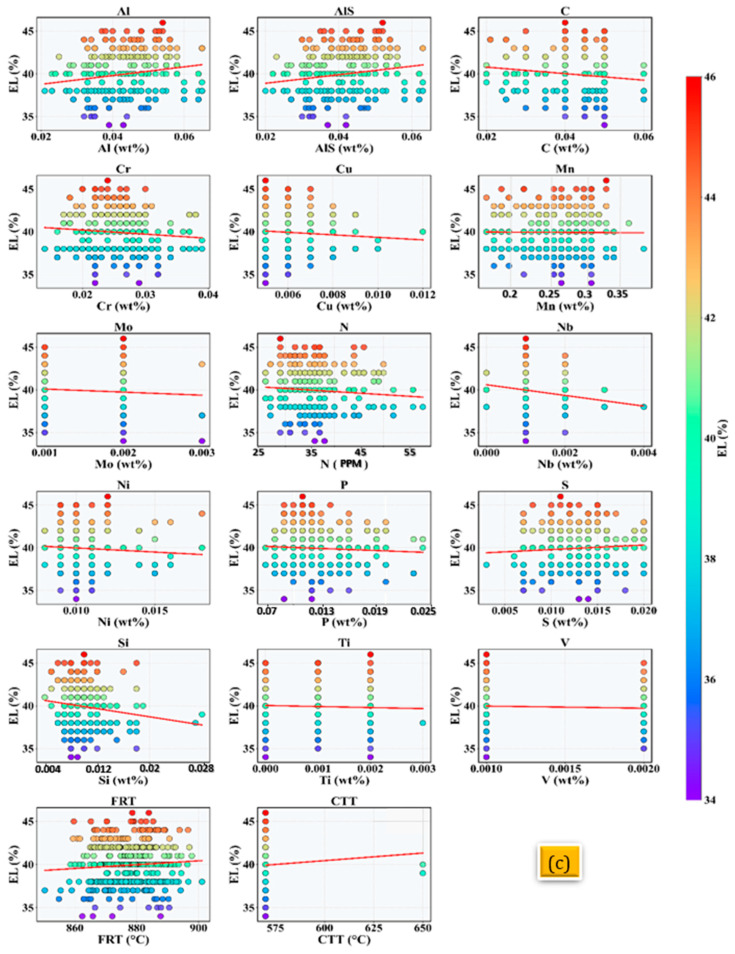
Effects of alloying elements and processing parameters on the (**a**) yield strength, (**b**) ultimate tensile strength, and (**c**) elongation.

From a total of 435 datasets, 335 sets were randomly selected to train the neural network model, whereas the remaining 100 sets were used for testing and validation. Figure 1a–c present the relationships between the mechanical properties YS, UTS, and El% and the key input features, including the elemental composition and processing parameters. The datasets encompass a wide range of alloy chemistries with a specific focus on alloying elements, such as Al, Cr, C, Mn, and N, as well as processing variables, such as FRT and CTT. The elemental contents were measured in atomic or weight percentages, highlighting their influence on mechanical behavior. Interstitial elements, such as carbon and nitrogen, play a crucial role in strengthening mechanisms, contributing positively to both strength and ductility, likely owing to solid solution strengthening and deformation mechanisms, such as TRIP or TWIP effects [7].

Similarly, Mn exhibits beneficial effects by stabilizing the austenitic phase and enhancing strain-hardening capacity. In contrast, elements such as aluminum and silicon tend to reduce elongation, possibly owing to the formation of hard intermetallic phases, whereas their effect on strength remains neutral to slightly positive. Chromium exhibits a mild positive effect on strength but has a less clear influence on ductility. Trace elements such as Mo, Nb, Ti, and V show limited compositional variation and correspondingly subtle effects; however, their role in precipitation strengthening cannot be reduced. Cu and P contributed marginally to the strengthening, with little impact on ductility. The processing parameters significantly influence the mechanical response. Higher coiling temperatures generally decrease both the strength and ductility, likely owing to coarser microstructures, whereas the effects of final rolling are more complex and less monotonic. Overall, these compositional and processing features serve as critical input variables for modeling the strength–ductility relationship, providing valuable guidance for alloy design and process optimization aimed at achieving the desired mechanical performance.

### 2.2. Neural Network Architecture and Training

**Figure 2:**
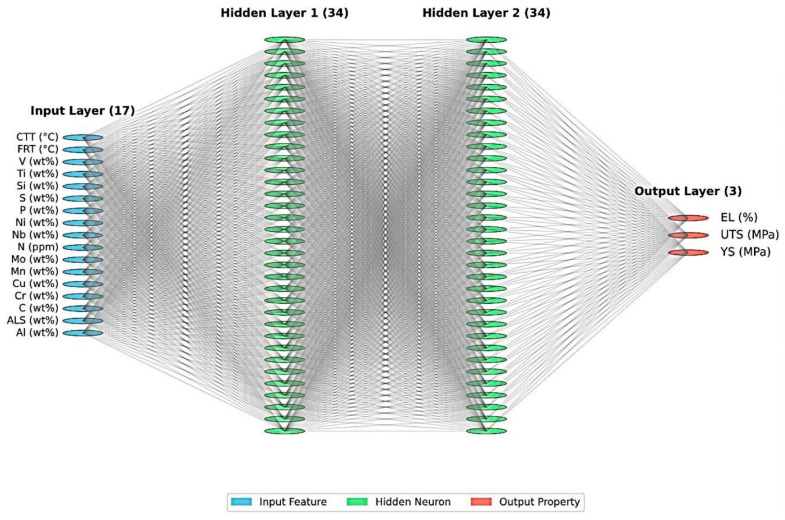
Schematic representation of the feed-forward neural network architecture.

A feed-forward neural network (FFNN) model was developed using a multilayer perceptron architecture. The network consisted of an input layer with 17 neurons (corresponding to the 15 composition elements plus the FRT and CTT), two hidden layers, and an output layer with three neurons (corresponding to the YS, the UTS, and the EL). Through extensive experimentation, it was found that the optimal neural network architecture comprises two hidden layers, with 34 neurons in each layer. This configuration provided the best balance between predictive accuracy and computational efficiency. The network architecture is illustrated in Figure 2. Prior to training, all input and output data were normalized to the range [0.1 to 0.9] using min-max scaling to enhance the training efficiency and prevent any bias due to the different magnitudes of the variables. This normalization was reversed during the prediction phase to obtain actual values of the mechanical properties. Neural network training was performed using a backpropagation algorithm with gradient descent optimization.

A total of 435 datasets were available, of which 335 were used for training and 100 were exclusively reserved for testing. No separate validation or cross-validation schemes was employed. Instead, model optimization was performed through extensive experimentation by varying the number of hidden neurons (15–40), learning rate (0.05–0.95), momentum term (0.05–0.95), and number of iterations (1000–200,000). The final architecture and hyperparameters were selected based on minimal training and test errors, as well as stable convergence to ensure a balance between model accuracy and generalization.

The learning rate (η) was set to 0.7, and momentum factor (α) was 0.6. These hyperparameters were selected after systematic trials with different combinations (learning rates from 0.1 to 0.9, and momentum factors from 0.1 to 0.9), where 0.7 and 0.6 provided the fastest convergence while avoiding local minima. The training process continued for 200,000 iterations, at which point the mean squared error (MSE) reached its optimum value of 0.014.

The activation function used in the hidden layers is a sigmoid function, which is defined as

f(x) = (e*^x^*− e^−*x*^)/(e*^x^*+ e^−*x*^) (1)

To determine the optimal ANN architecture for predicting YS, UTS, and EL, a series of systematic experiments were conducted by varying the network parameters. Architectures with one, two, and three hidden layers were tested, with the number of neurons in each layer ranging from 17 to 50. The best performance was achieved by using a network with two hidden layers, each containing 34 neurons. The learning rate (η) and momentum term (α) were varied independently from 0.05 to 0.95 in steps of 0.05 to evaluate their impact on the training efficiency and convergence behavior. The optimal combination was found to have a learning rate of 0.7 and a momentum term of 0.6. The training process was iterated for up to 200,000 cycles, beyond which no further reduction in the error was observed. The final model achieved a minimum mean squared error (MSE) of 0.014, indicating a high level of accuracy and convergence stability. A sigmoid activation function is employed in the output layer to ensure bounded nonlinear predictions of the target mechanical properties.

### 2.3. Model Evaluation and Analysis of Processing Parameters

The performance of the trained neural network model was evaluated using 100 test datasets that were not used in the training process. Prediction accuracy was assessed by calculating the mean percentage error (MPE) between the predicted and actual YS, UTS, and EL values. The MPE is defined as

MPE = (1/n) × Σ| (predicted–actual)/actual| × 100%(2)

where n is the number of test samples. In addition, the performance of the model was visually inspected by plotting the predicted values against the actual values for each mechanical property. To investigate the influence of the processing parameters on the mechanical properties, hypothetical alloy compositions were created by systematically varying the FRT and CTT, while maintaining a constant chemical composition. Specifically, the FRT was varied from 850 °C to 901 °C in 5 °C increments (13 data points), whereas the CTT was varied from 570 °C to 650 °C in 10 °C increments (nine data points). This approach allowed us to isolate the effects of these critical processing parameters on the mechanical properties of steel strips.

The selection of these processing parameter ranges is based on established metallurgical principles and industrial practices. The FRT range (850–901 °C) window corresponds to the optimal austenite-conditioned zone for low-carbon steels. The lower limit (850 °C) ensures adequate plastic deformation of austenite while avoiding excessive work hardening, which could lead to inhomogeneous microstructures. The upper limit (901 °C) prevented excessive austenite grain growth, which adversely affected the final mechanical properties. This range encompasses the critical temperatures at which controlled austenite-to-ferrite transformation occurs, which directly influences the final ferrite grain size and mechanical properties [12,18,21,24].

The CTT range (570–650 °C) represents the typical industrial practices for low-carbon hot-rolled strips. The lower limit (570 °C) was strategically chosen to be above the pearlite transformation nose to prevent uncontrolled pearlite formation while allowing controlled ferrite formation. The upper limit (650 °C) prevents excessive ferrite grain coarsening and maintains the desired strength–ductility balance. Coiling within this range ensures the optimal precipitation kinetics of microalloying elements when present [12,18,25]. The 5 °C increments for FRT provided sufficient resolution to capture the rapid microstructural changes occurring during the γ→α transformation, particularly the nucleation and growth kinetics of ferrite. The 10 °C increments for CTT are appropriate given that the coiling temperature effects are generally less pronounced and more gradual compared to the finish rolling temperature effects. The processing parameters used in this study, including FRT (868 °C) and CTT (570 °C) for sample 8 validation, represent industrially feasible operating conditions within standard hot-strip mill ranges (850–901 °C for FRT, 570–650 °C for CTT), ensuring the practical applicability of the model predictions.

## 3. Results

### 3.1. Model Performance on Test Data

**Figure 3:**
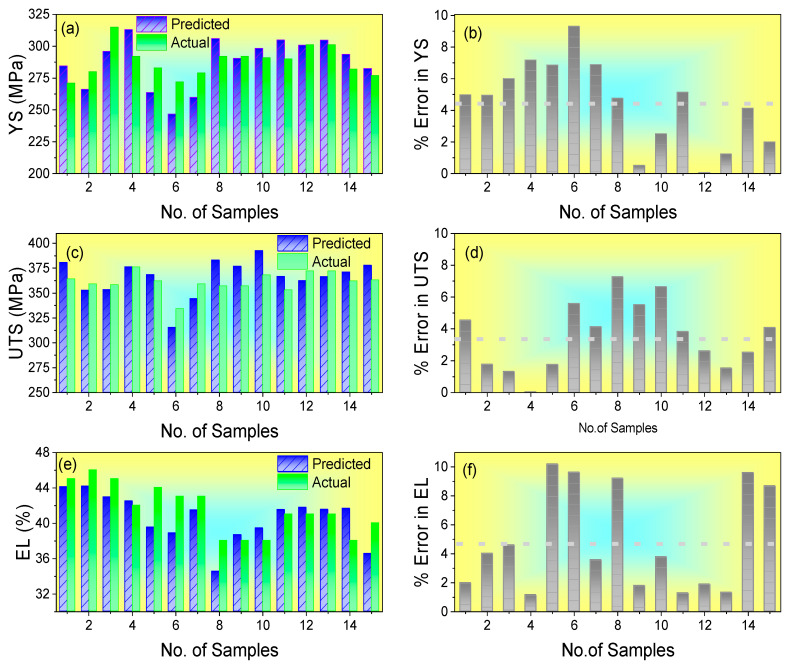
Comparison of predicted and actual mechanical properties for 15 test samples: (**a**,**b**) Yield Strength (YS), (**c**,**d**) Ultimate Tensile Strength (UTS), and (**e**,**f**) Elongation (EL), with corresponding percentage errors. Adapted from our previous work [26].

The performance of the FFNN model on the test data is illustrated in Figure 3, which compares the predicted and actual values of YS, UTS, and EL for ten randomly selected samples from the 100 test data points [26]. These samples were selected to provide a representative view of the predictive accuracy of the model for different mechanical properties.

The results demonstrated excellent agreement between the predicted and actual values. The mean percentage errors for YS, UTS, and EL were calculated as 4.44%, 3.54%, and 4.84%, respectively. This level of accuracy is highly satisfactory for practical applications in steel manufacturing and indicates that the neural network model effectively captures the complex relationships between composition, processing parameters, and mechanical properties. More than 90% of the predictions were within ±5% of the actual values, which is consistent with or better than previous studies on the prediction of mechanical properties of steels using neural networks (ex. Table 2). This accuracy level is sufficient for industrial applications, where a prediction error of less than 5% is generally considered acceptable for quality control and process optimization purposes.

### 3.2. Effect of Processing Parameters on Mechanical Properties

**Figure 4:**
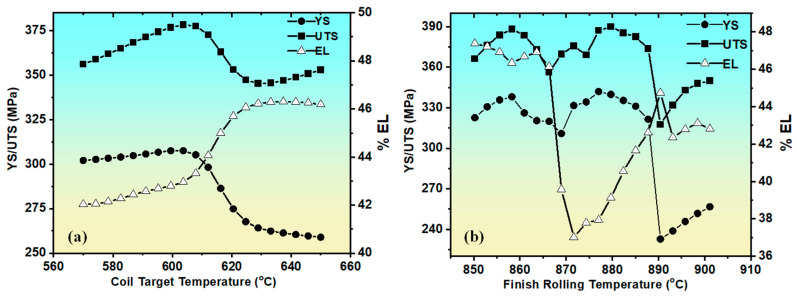
Effect of the variation of (**a**) coil target temperature and (**b**) finish rolling temperature and on mechanical properties. Adapted from our previous work [26].

One of the key objectives of this study was to understand the influence of the processing parameters (FRT and CTT) on the mechanical properties of the hot-rolled steel strips. Parametric studies were conducted using systematic variations: FRT from 850 to 901 °C (5 °C increments) and CTT from 570 to 650 °C (10 °C increments), maintaining a constant chemical composition throughout the analysis. Figure 4 illustrates the effects of varying the FRT and CTT on the YS, UTS, and EL based-on model-generated predictions for hypothetical variations in individual input parameters [26]. The results revealed that FRT has a more complex influence on mechanical properties than CTT. The results revealed that FRT has a more complex influence on mechanical properties than CTT. Within the investigated FRT range (850–901 °C), the yield strength exhibited non-monotonic behavior, initially increasing from 850 °C to approximately 880 °C, reaching a maximum at approximately 885 °C, and then gradually decreasing toward 901 °C. This complex behavior can be attributed to the fact that FRT affects multiple microstructural features simultaneously, including the volume fraction and morphology of proeutectoid ferrite, austenite grain size prior to transformation, ferrite grain size and distribution, texture development during rolling, and precipitation kinetics of carbides and nitrides.

The CTT range (570–650 °C) exhibited more predictable trends, with generally decreasing strength properties and increasing ductility as the coiling temperature increased. In contrast, the CTT is below the eutectoid temperature, resulting in smaller variations in the microstructure and, consequently, less pronounced effects on the mechanical properties compared to FRT. The 5 °C resolution for FRT was crucial in capturing the sharp transitions in mechanical properties, particularly around 880–890 °C where the maximum strength values were observed. The 10 °C resolution for the CTT adequately captured the gradual property changes associated with coiling temperature variations.

To further validate the capability of the model to predict the effects of the processing parameters, we compared the predicted mechanical properties with the actual values for a specific steel composition when varying the FRT and CTT. Table 2 presents the results for sample 8 from the dataset. The results indicated that the model predictions were in good agreement with the actual values, with deviations within an acceptable range of 5%. This confirms the ability of the model to accurately predict the effects of the processing parameters on the mechanical properties, which is valuable for process optimization in steel manufacturing [18,24,25,27].

### 3.3. Importance of Carbon and Manganese

**Figure 5:**
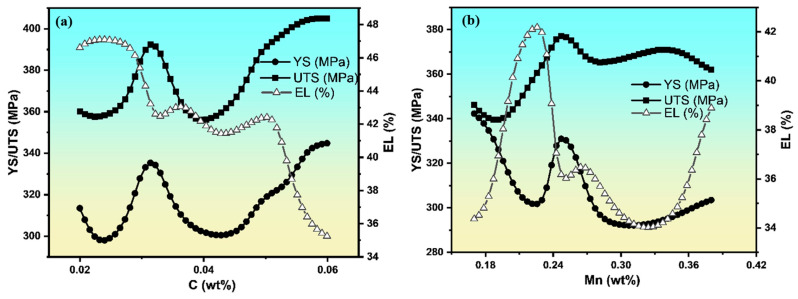
Variation in mechanical properties with alloying element content: (**a**) effect of C content (0.02–0.06 wt%) on YS, UTS, and EL; (**b**) effect of Mn content (0.17–0.38 wt%) on YS, UTS, and EL.

To gain an insight into the relative importance of different input parameters in determining mechanical properties, we performed a sensitivity analysis on the trained neural network model. This involved systematically varying each input parameter while keeping the others constant, and observing the corresponding changes in the predicted mechanical properties. The results of the sensitivity analysis revealed that, among the composition elements, C and Mn had the most significant influence on all three mechanical properties. The carbon content has a pronounced impact on both the strength and ductility, as illustrated in Figure 5a. The YS exhibited significant fluctuations with carbon content, ranging from approximately 300 to 340 MPa across the investigated carbon range of 0.02–0.06 wt%. This nonmonotonic behavior can be attributed to the complex interplay between solid solution strengthening, precipitation hardening, and microstructural evolution.

The UTS exhibited an oscillatory trend between 358 and 405 MPa, with peak values occurring at intermediate carbon contents. This reflects the balance between the carbon retained in the matrix and its role in the carbide formation. Figure 5b illustrates the effect of the Mn content on the mechanical properties. Notably, YS and UTS converge at Mn levels below ~0.18 wt%, indicating reduced strain hardening capacity. At such a low Mn content, the alloy lacks sufficient solid solution strengthening and austenite stability, leading to early plastic instability and a brittle-like response. The corresponding decrease in EL further supports this interpretation, demonstrating the ability of the ANN model to capture meaningful metallurgical trends.

Between 0.17 and 0.38 wt% Mn, the mechanical behavior becomes more complex because of the dual role of Mn in solid solution strengthening and transformation kinetics. The YS varies from ~290 to 340 MPa, and the UTS varies from ~340 to 380 MPa. EL generally shows an inverse relationship with strength, which is consistent with the typical strength–ductility trade-off in steel. The elongation declined from ~47% at a low carbon content to ~35% at higher levels, with intermediate fluctuations that mirror strength trends [10,28,29].

The sensitivity curves in Figure 4 are based on ANN-generated predictions by varying individual input parameters (e.g., C and Mn) while keeping the others constant. Since these values were not derived from repeated experimental trials, standard deviation bands or 95% confidence intervals could not be applied.

The ultimate tensile strength demonstrated a similar behavior, with values ranging from 295 to 340 MPa, showing maximum strength at intermediate manganese levels. The elongation response to manganese content reveals an interesting bimodal behavior, with high ductility (>40%) at both low and high manganese contents, and reduced ductility (34–36%) in the intermediate range around 0.25–0.32 wt% Mn.

The predicted values shown in Table 2 for these hypothetical alloys closely matched the actual measured properties (YS = 302 MPa, UTS = 356 MPa, EL = 42%), demonstrating the predictive reliability of the model. For instance, varying C to 0.04 wt% resulted in YS = 301.5 MPa, UTS = 356.3 MPa, and EL = 41.8%, while adjusting Mn to 0.22 wt% yielded YS = 301.8 MPa, UTS = 357.3 MPa, and EL = 42.1%. Similarly, modifying the FRT to 868 °C predicted YS of 313 MPa and UTS of 365 MPa, reflecting an increase in strength with a marginal decrease in ductility. The predicted EL remained within ±0.2–0.5% of the actual value across all simulations, confirming the sensitivity and accuracy of the ANN model in capturing the effects of both compositional and processing changes. These findings confirm the capability of the model to perform virtual alloy trials, enabling the targeted optimization of steel properties with minimal experimental effort.

The industrial feasibility of the processing parameters used in this validation study is crucial for their practical implementation. The FRT of 868 °C and CTT of 570 °C employed for sample 8 represent the standard operating conditions in modern hot strip mills, ensuring that the model’s predictive capabilities are directly applicable to real-world manufacturing scenarios. These temperature ranges are routinely achieved in industrial settings and are aligned with energy-efficient processing practices, while maintaining consistent product quality. This phenomenon is attributed to the competing effects of Mn on grain refinement, austenite stability, and precipitation behavior [30,31]. These complex relationships suggest that both carbon and Mn interact with other microstructural features and processing parameters to influence the final mechanical properties. This non-monotonic behavior indicates that optimal property combinations may be achieved through the careful control of these alloying elements within specific compositional windows. This finding is consistent with established metallurgical principles, as these elements strongly affect the microstructure and strengthening mechanisms of low-carbon steels [7].

**Figure 6:**
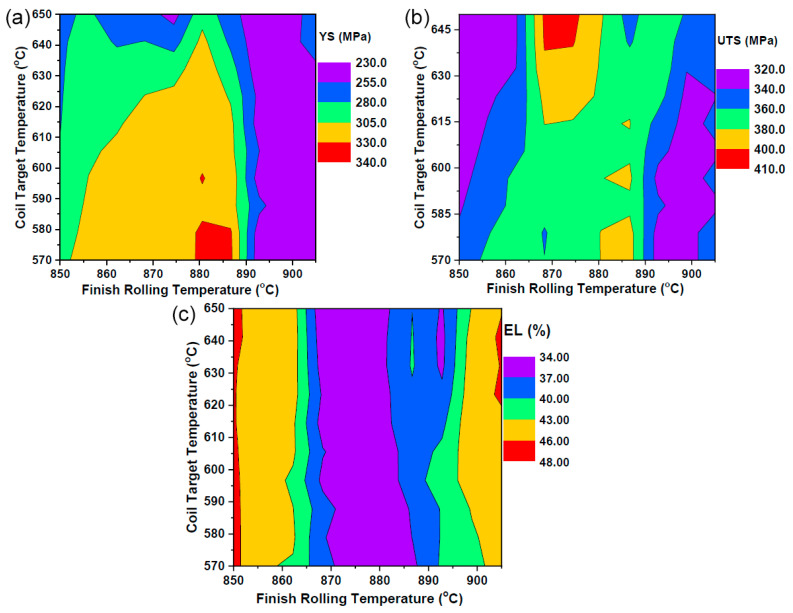
Combined effect of finish rolling temperature (FRT) and coil target temperature (CTT) on mechanical properties: (**a**) YS, (**b**) UTS, and (**c**) EL. Adapted from our previous work [26].

Among the processing parameters, the FRT demonstrated a more pronounced effect on the mechanical properties than the CTT, particularly on the YS and UTS. This dominance can be attributed to FRT’s significant influence of FRT on austenite-to-ferrite transformation kinetics and the resulting microstructural development, which directly governs the evolution of mechanical properties [32,33,34]. The contour maps in Figure 6 were generated using the systematic parameter variations described above (FRT: 850–901 °C in steps of 5 °C; CTT: 570–650 °C in steps of 10 °C), creating a comprehensive processing map with unique processing conditions [26]. The color legends in the contour plots are displayed in descending order based on the default settings of the plotting software. The color scale was consistent across all plots for comparative analysis. For the YS, the contour map shows distinct regions of property optimization, with maximum values (330–340 MPa) achieved within the narrow processing window of 875–890 °C FRT combined with 580–600 °C CTT. This optimal zone represented approximately 15% of the total processing space investigated, highlighting the critical importance of precise process control. Lower finish rolling temperatures generally produce reduced yield strength, regardless of the coil target temperature, whereas higher FRT values (>900 °C) also show diminished strengthening effects. The UTS response exhibited similar but more pronounced variations, concentrated in a narrow processing window of approximately 880 °C FRT and 580–590 °C CTT. Notably, the peak values were concentrated around 880 °C FRT and 585 °C CTT, with the optimal processing window being narrower than that for the yield strength. Deviations of ±10 °C in the FRT or ±15 °C in the CTT from optimal conditions resulted in a 15–25 MPa reduction in the UTS, indicating the detrimental effects of excessive thermal exposure on the tensile properties.

The elongation demonstrates an inverse relationship with the strength properties, which is consistent with the typical steel behavior. The highest ductility values (46–48%) were achieved at processing extremes, requiring either low FRT (850–860 °C) with high CTT (630–650 °C) or high FRT (890–901 °C) and low CTT (570–590 °C). This bimodal distribution confirms the fundamental strength-ductility trade-off and provides clear guidance for process design based on target properties [35,36]. Contour analysis revealed that optimal mechanical property combinations require precise control of both thermal parameters within narrow processing windows, emphasizing the critical nature of thermomechanical processing in achieving target steel properties.

### 3.4. Comparison with Existing Models

To evaluate the performance of our FFNN model in a broader context, we compared its predictions with those of existing models. Although direct comparisons are challenging owing to the differences in steel compositions and processing conditions, our model demonstrated comparable or superior performance in terms of prediction accuracy. For instance, Singh and Bhadeshia [16] reported prediction errors in the range of 5–10% for the mechanical properties of low-alloy steels using neural networks, which is higher than the errors achieved by our model (3.54–4.84%). Similarly, Chang and Bhadeshia [17] achieved prediction accuracies comparable to those of our model but with a more limited set of input parameters. More recently, Mathur and Singh [4] reported mean percentage errors of approximately 5–7% for YS and UTS predictions in hot-rolled steel plates, which is slightly higher than our model’s error rates. The enhanced performance of our model can be attributed to several factors such as the comprehensive inclusion of 15 composition elements along with key processing parameters (FRT and CTT), an optimized neural network architecture with two hidden layers and 34 neurons per layer, and a large and diverse dataset (435 samples) used for training and testing.

### 3.5. Graphical User Interface for Mechanical Properties Prediction

**Figure 7:**
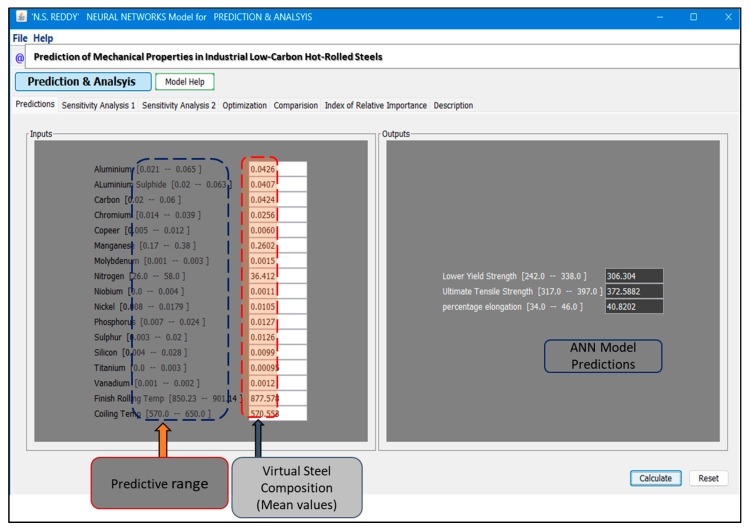
GUI for estimating the mechanical properties by manually entering steel composition and processing parameters. The developed ANN model features a user-friendly GUI designed to predict the YS, UTS, and EL of low-carbon hot-rolled steel strips.

A graphical user interface (GUI) was designed and implemented to enhance the practical applicability of the developed neural network model. The ANN model was trained using a specialized neural network software, and the GUI was developed using Java to enable cross-platform compatibility and efficient deployment. As shown in Figure 7, the GUI enables the accurate prediction of mechanical properties for infinite combinations of the 17 input parameters: 15 composition elements, along with the finish rolling temperature (FRT) and coil target temperature (CTT). The developed graphical user interface (GUI) enables users to efficiently predict mechanical properties, such as lower yield strength (YS), ultimate tensile strength (UTS), and percentage elongation (%EL), with a single click, allowing for the rapid exploration of various compositional and processing conditions. Users can input specific values for 15 composition elements and processing parameters within their respective ranges, and the “Predict” button generates corresponding YS, UTS, and %EL values. The GUI also supports sensitivity analysis to evaluate the influence of individual variables and their interactions, providing valuable insights into the complex nonlinear relationships that govern the mechanical behavior of low-carbon hot-rolled steel. In addition, the GUI offers visualization tools that allow users to generate graphs showing the impact of key parameters, such as carbon content, manganese content, FRT, and CTT, on mechanical properties. This functionality is particularly useful for optimizing the alloy design and processing parameters to achieve the target performance while minimizing experimental effort.

Using this tool, the ANN model predicted the mechanical properties of a data point constructed from the mean values of all input parameters. The predicted values—306.30 MPa for YS, 372.59 MPa for UTS, and 40.82% for %EL—were all higher than the corresponding mean values in the test dataset (293.68 MPa, 365.35 MPa, and 39.93%, respectively). These predictions, based on the network’s trained synaptic weights, demonstrate the model’s ability to generalize and accurately capture complex input–output relationships. Consequently, ANN-based tools significantly reduce the time, cost, and material requirements traditionally associated with experimental alloy development. The GUI and its source code are available upon request from the researchers and industry professionals. Basic training and user support will be provided to encourage broader adoption within the steel manufacturing community and academic institutions, thereby promoting practical applications and collaborative research.

## 4. Conclusions

In this study, we developed and validated a feed-forward neural network model for predicting the mechanical properties (YS, UTS, and EL) of low-carbon hot-rolled steel strips based on chemical composition and processing parameters. The key findings and conclusions are summarized below.

1. The optimized FFNN model with two hidden layers (34 neurons each) demonstrated excellent predictive capability, with mean percentage errors of 4.44%, 3.54%, and 4.84% for YS, UTS, and EL, respectively.
2. The finish rolling temperature (FRT) showed a more complex influence on the mechanical properties than the coil target temperature (CTT), which can be attributed to the broader effects of the FRT on the microstructural evolution during hot rolling.
3. Among the composition elements, carbon and manganese were identified as the most influential factors affecting the mechanical properties, which aligns with established metallurgical principles.
4. The model successfully captured the combined effects of composition and processing parameters on the mechanical properties, thereby providing a valuable tool for alloy design and process optimization in steel manufacturing.
5. The prediction accuracy of our model is comparable or superior to that of the existing models in the literature, highlighting the benefits of incorporating a comprehensive set of input parameters and optimizing the neural network architecture.
6. The processing parameters employed in the model validation are industrially feasible and representative of actual manufacturing conditions, ensuring practical applicability in real-world steel production environments.

The developed neural network model offers significant practical benefits for steel manufacturers by enabling the accurate prediction of mechanical properties based on the composition and processing parameters. Future work should focus on extending the model to include additional processing parameters and microstructural features to enhance its predictive capabilities further. The integration of this model with physical metallurgy principles could also lead to an improved understanding of the structure–property relationships in hot-rolled steel strips.

## Figures and Tables

**Table 1:** Composition and processing parameter ranges for hot-rolled steel strips.

Parameter	Minimum	Maximum	Average	Std. Deviation
Al (wt%)	0.021	0.065	0.043	0.011
ALS (wt%)	0.020	0.063	0.041	0.010
C (wt%)	0.020	0.060	0.040	0.008
Cr (wt%)	0.014	0.039	0.026	0.006
Cu (wt%)	0.005	0.012	0.008	0.002
Mn (wt%)	0.170	0.380	0.275	0.052
Mo (wt%)	0.001	0.003	0.002	0.001
N (ppm)	26	58	42	8
Nb (wt%)	0.000	0.004	0.002	0.001
Ni (wt%)	0.008	0.018	0.013	0.002
P (wt%)	0.007	0.024	0.015	0.004
S (wt%)	0.003	0.020	0.011	0.004
Si (wt%)	0.004	0.028	0.016	0.006
Ti (wt%)	0.000	0.003	0.001	0.001
V (wt%)	0.001	0.002	0.001	0.0004
FRT (°C)	850.23	901.14	875.68	12.73
CTT (°C)	570	650	610	20.41
YS (MPa)	242	338	290	24.12
UTS (MPa)	317	397	357	20.15
EL (%)	34	46	40	3.02

**Table 2:** Comparison of actual and predicted properties of hot-rolled steel strips for sample 8 using industrially feasible processing parameters. The FRT (868 °C) and CTT (570 °C) values were within the standard industrial operating ranges for low-carbon hot-strip mills.

System	YS (MPa)	UTS (MPa)	EL (%)
Actual	302	356	42
Hypothetical alloy based on C (0.04) (wt%)	301.5	356.3	41.8
Hypothetical alloy based on Mn (0.22) (wt%)	301.8	357.3	42.1
Hypothetical alloy based on FRT (868 °C)	313	365	41.8
Hypothetical alloy based on CTT (570 °C)	303	358	42.5

## Data Availability

All data are available in the Supplementary Materials.

## Supplementary Materials

The following supporting information can be downloaded at: https://www.mdpi.com/article/10.3390/ma18132966/s1, Supplementary datal (Appendix: Hot-rolled steel).

## Abbreviations

The following abbreviations are used in this manuscript:

FRT	Finish rolling temperature
CTT	Coil target temperature

## References

[1] Bhadeshia H.K.D.H. (2009). Neural Networks and Information in Materials Science. Stat. Anal. Data Min..

[2] Bhadeshia H. (2006). Neural Networks in Materials Science: The Importance of Uncertainty.

[3] Sha W., Edwards K.L. (2007). The Use of Artificial Neural Networks in Materials Science Based Research. Mater. Des..

[4] Ishtiaq M., Tiwari S., Nagamani M., Kang S.-G., Reddy N.G. (2025). Data-Driven ANN-Based Predictive Modeling of Mechanical Properties of 5Cr-0.5Mo Steel: Impact of Composition and Service Temperature. Crystals.

[5] Reséndiz-Flores E.O., Altamirano-Guerrero G., Costa P.S., Salas-Reyes A.E., Salinas-Rodríguez A., Goodwin F. (2021). Optimal Design of Hot-Dip Galvanized DP Steels via Artificial Neural Networks and Multi-Objective Genetic Optimization. Metals.

[6] Narayana P.L., Tiwari S., Maurya A.K., Ishtiaq M., Park N., Reddy N.G. (2025). Quantitative and Qualitative Analysis of Atmospheric Effects on Carbon Steel Corrosion Using an ANN Model. Metals.

[7] Guessasma S., Montavon G., Coddet C. (2004). Neural Computation to Predict In-Flight Particle Characteristic Dependences From Processing Parameters in the APS Process. J. Therm. Spray Technol..

[8] Ishtiaq M., Tiwari S., Panigrahi B.B., Seol J.B., Reddy N.S. (2024). Neural Network-Based Modeling of the Interplay between Composition, Service Temperature, and Thermal Conductivity in Steels for Engineering Applications. Int. J. Thermophys..

[9] Dutta R.K., Petrov R., Delhez R., Hermans M.J.M., Richardson I.M., Böttger A.J. (2013). The Effect of Tensile Deformation by in Situ Ultrasonic Treatment on the Microstructure of Low-Carbon Steel. Acta Mater..

[10] Mukherjee M., Singh S.B. (2009). Artificial Neural Network: Some Applications in Physical Metallurgy of Steels. Mater. Manuf. Process..

[11] Feng X., Gao X., Luo L. (2021). A ResNet50-Based Method for Classifying Surface Defects in Hot-Rolled Strip Steel. Mathematics.

[12] Mandal A., Ghosh A., Chakrabarti D., Davis C. (2021). Effect of Coiling Temperature on Impact Toughness of Hot Rolled Ultra-High-Strength Multiphase Steel Strips. Mater. Sci. Eng.—Struct. Mater. Prop. Microstruct. Process..

[13] Corfar D.-A., Tsavdaridis K.D. (2022). A Comprehensive Review and Classification of Inter-Module Connections for Hot-Rolled Steel Modular Building Systems. J. Build. Eng..

[14] Rademacher D., Ochojski W., Lorenc W., Kożuch M. (2018). Advanced Solutions with Hot-Rolled Sections for Economical and Durable Bridges. Steel Constr..

[15] Saastamoinen A., Kaijalainen A., Porter D., Suikkanen P., Yang J.-R., Tsai Y.-T. (2018). The Effect of Finish Rolling Temperature and Tempering on the Microstructure, Mechanical Properties and Dislocation Density of Direct-Quenched Steel. Mater. Charact..

[16] Zhao Z., Tang J., Tariq N.u.H., Liu H., Liu H., Ren Y., Tong M., Yin L., Du H., Wang J. (2020). Effect of Rolling Temperature on Microstructure and Mechanical Properties of Ti/Steel Clad Plates Fabricated by Cold Spraying and Hot-Rolling. Mater. Sci. Eng.—Struct. Mater. Prop. Microstruct. Process..

[17] Guo B., Fan L., Wang Q., Fu Z., Wang Q., Zhang F. (2016). Effect of Finish Rolling Temperature on the Microstructure and Tensile Properties of Nb–Ti Microalloyed X90 Pipeline Steel. Metals.

[18] Xue J., Zhao Z., Bin C., Liu X., Wu H., Li H., Xiong W. (2019). Effects of Rolling and Coiling Temperature on the Microstructure and Mechanical Properties of Hot-Rolled High Strength Complex Phase Steel. Mater. Res. Express.

[19] Singh S.B., Bhadeshia H.K.D.H. (1998). Estimation of Bainite Plate-Thickness in Low-Alloy Steels. Mater. Sci. Eng.—Struct. Mater. Prop. Microstruct. Process..

[20] Chang L.C., Bhadeshia H.K.D.H. (1996). Microstructure of Lower Bainite Formed at Large Undercoolings below Bainite Start Temperature. Mater. Sci. Technol..

[21] Wang X., Li X., Yuan H., Zhou N., Wang H., Zhang W., Ji Y. (2024). Prediction and Analysis of Mechanical Properties of Hot-Rolled Strip Steel Based on an Interpretable Machine Learning. Mater. Today Commun..

[22] Wu H., Zhang B., Li Z. (2024). Small Sample-Oriented Prediction Method of Mechanical Properties for Hot Rolled Strip Steel Based on Model Independent Element Learning. IEEE Access.

[23] Fang W., Huang J.X., Peng T.X., Long Y., Yin F.X. (2024). Machine Learning-Based Performance Predictions for Steels Considering Manufacturing Process Parameters: A Review. J. Iron Steel Res. Int..

[24] NBC Engineers (2008). The Complete Technology Book on Steel and Steel Products (Fasteners, Seamless Tubes, Casting, Rolling of Flat Products & Others): How to Start Steel Rolling Mill, Iron and Steel Making by-Products, Manufacturing of Steel, Manufacturing Process for Steel Products, Metal Fasteners Manufacturing, Mill Automation for Pipe and Tubing Production, Modern Rolling Plant, Modern Small and Cottage Scale Industries, Most Profitable Steel Business Ideas, New Small Scale Ideas in Steel Rolling Industry, Opportunity Steel Rolling Mill, Plate Mill, Production of Welded Pipe, Profitable Small and Cottage Scale Industries.

[25] Seetharaman S. (2005). Fundamentals of Metallurgy.

[26] Reddy N.S., Panigrahi B.B., Krishnaiah J. (2013). Modeling Mechanical Properties of Low Carbon Hot Rolled Steels.

[27] Paupler P.G.E. (1988). Dieter. Mechanical Metallurgy. 3rd Ed., Mc Graw-Hill Book Co., New York 1986. XXIII + 751 p., DM 138.50, ISBN 0–07–016893–8. Cryst. Res. Technol..

[28] Laz’ko V.G., Nikitin V.N., Karchevskaya N.I. (1986). Effect of Carbon Content on the Structure and Mechanical Properties of High-Strength Weldable Steel 03G4N2MAF. Met. Sci. Heat Treat..

[29] Goritskii V.M., Shneiderov G.R., Guseva I.A. (2019). Effect of Chemical Composition and Structure on Mechanical Properties of High-Strength Welding Steels. Metallurgist.

[30] Khorrami M., Hanzaki A.Z., Abedi H.R., Moallemi M., Mola J., Chen G. (2021). On the Effect of Mn-Content on the Strength-Ductility Balance in Ni-Free High N Transformation Induced Plasticity Steels. Mater. Sci. Eng.—Struct. Mater. Prop. Microstruct. Process..

[31] Song R., Ponge D., Raabe D. (2005). Influence of Mn Content on the Microstructure and Mechanical Properties of Ultrafine Grained C-Mn Steels. Isij Int..

[32] Olalla V.C., Petrov R., Thibaux P., Liebeherr M., Gurla P., Kestens L.A. (2012). Influence of Rolling Temperature and Cooling Rate on Microstructure and Properties of Pipeline Steel Grades. Mater. Sci. Forum.

[33] Huang Y., Han J., Liu W., Li F., Zhao A., Liu Y. (2020). Effect of the Final Rolling Temperature on the Precipitation Behavior and Toughening Mechanism of Nanoparticles in Ferritic Steel. J. Mater. Eng. Perform..

[34] Kim K.H., Hwang N.-M., Lee B.-J., Yoon J.-K. (2005). Effects of the Finish Rolling Temperature on Mechanical Properties and Microstructure Evolution of Line Pipe Steel. Mater. Sci. Forum.

[35] An Y.F., Chen X.P., Mei L., Tan X., Ren P., Zhang X.Y., Cao W.Q. (2024). Overcoming the Strength-Ductility Trade-off Dilemma in Austenitic Lightweight Steel via Stepwise Controllable Intragranular Dual Nanoprecipitation. Mater. Sci. Eng. A.

[36] Li X.T., Liu R., Hou J., Zhang Z.J., Zhang Z.F. (2025). Trade-off Model for Strength-Ductility Relationship of Metallic Materials. Acta Mater..

