# Supplementary material for: Mechanical Property Prediction of Industrial Low-Carbon Hot-Rolled Steels Using Artificial Neural Networks
Source: Materials (Basel). 2025 Jun 23;18(13):2966. doi: 10.3390/ma18132966 (PMC12250638; doi:10.3390/ma18132966)
Supplement: Supplementary file 1 [file materials-18-02966-s001.zip › materials-3688078-supplementary.pdf]

Sl. No.	Al	AlS	C	Cr	Cu	Mn	Mo	N
1	0.04	0.039	0.05	0.029	0.006	0.29	0.001	36
2	0.032	0.03	0.035	0.024	0.006	0.19	0.002	58
3	0.045	0.044	0.035	0.03	0.007	0.24	0.001	35
4	0.042	0.041	0.05	0.029	0.006	0.3	0.001	42
5	0.042	0.04	0.045	0.031	0.006	0.21	0.001	38
6	0.038	0.036	0.045	0.024	0.006	0.24	0.001	43
7	0.05	0.048	0.04	0.026	0.008	0.28	0.001	43
8	0.034	0.032	0.04	0.028	0.006	0.22	0.001	45
9	0.049	0.047	0.04	0.026	0.005	0.28	0.002	33
10	0.046	0.044	0.04	0.029	0.006	0.32	0.002	49
11	0.046	0.044	0.04	0.029	0.006	0.32	0.002	49
12	0.033	0.031	0.04	0.024	0.005	0.23	0.002	32
13	0.033	0.031	0.04	0.024	0.005	0.23	0.002	32
14	0.033	0.031	0.04	0.024	0.005	0.23	0.002	32
15	0.049	0.047	0.04	0.026	0.005	0.28	0.002	33
16	0.049	0.047	0.04	0.026	0.005	0.28	0.002	33
17	0.049	0.047	0.04	0.026	0.005	0.28	0.002	33
18	0.046	0.044	0.04	0.029	0.006	0.32	0.002	49
19	0.046	0.044	0.04	0.029	0.006	0.32	0.002	49
20	0.046	0.044	0.04	0.029	0.006	0.32	0.002	49
21	0.065	0.063	0.05	0.028	0.005	0.27	0.002	27
22	0.065	0.063	0.05	0.028	0.005	0.27	0.002	27
23	0.037	0.035	0.03	0.027	0.005	0.22	0.001	45
24	0.045	0.043	0.05	0.028	0.005	0.25	0.001	37
25	0.043	0.041	0.05	0.023	0.006	0.29	0.001	34
26	0.037	0.035	0.05	0.03	0.005	0.3	0.002	34
27	0.038	0.036	0.06	0.028	0.008	0.34	0.002	32
28	0.039	0.037	0.045	0.025	0.007	0.26	0.001	36
29	0.038	0.036	0.05	0.021	0.007	0.3	0.001	40
30	0.038	0.036	0.05	0.021	0.007	0.3	0.001	40
31	0.038	0.036	0.05	0.021	0.007	0.3	0.001	40
32	0.033	0.031	0.04	0.032	0.006	0.3	0.001	30
33	0.037	0.035	0.04	0.014	0.006	0.17	0.002	50
34	0.037	0.035	0.04	0.014	0.006	0.17	0.002	50
35	0.05	0.048	0.045	0.019	0.005	0.23	0.002	52
36	0.05	0.048	0.045	0.019	0.005	0.23	0.002	52
37	0.05	0.048	0.045	0.019	0.005	0.23	0.002	52
38	0.05	0.048	0.045	0.019	0.005	0.23	0.002	52
39	0.042	0.04	0.045	0.028	0.006	0.26	0.002	38
40	0.042	0.04	0.045	0.028	0.006	0.26	0.002	38
41	0.049	0.047	0.03	0.025	0.006	0.22	0.002	32
42	0.049	0.047	0.03	0.025	0.006	0.22	0.002	32
43	0.038	0.036	0.03	0.024	0.006	0.26	0.002	40
44	0.056	0.054	0.04	0.024	0.008	0.28	0.003	37
45	0.038	0.036	0.04	0.022	0.006	0.25	0.002	42
46	0.04	0.038	0.04	0.019	0.005	0.24	0.002	50
47	0.04	0.038	0.045	0.02	0.006	0.22	0.002	44
48	0.04	0.038	0.04	0.019	0.005	0.24	0.002	50
49	0.038	0.036	0.04	0.022	0.006	0.25	0.002	42

50	0.04	0.038	0.037	0.022	0.005	0.25	0.002	36
51	0.046	0.044	0.045	0.026	0.005	0.29	0.002	36
52	0.047	0.045	0.04	0.022	0.005	0.26	0.001	37
53	0.033	0.031	0.03	0.027	0.009	0.22	0.002	48
54	0.036	0.034	0.02	0.03	0.007	0.3	0.002	30
55	0.046	0.044	0.05	0.031	0.006	0.31	0.002	37
56	0.036	0.035	0.05	0.024	0.006	0.27	0.001	34
57	0.035	0.034	0.04	0.026	0.006	0.25	0.001	33
58	0.044	0.042	0.04	0.032	0.006	0.22	0.001	34
59	0.044	0.042	0.04	0.032	0.006	0.22	0.001	34
60	0.023	0.022	0.027	0.036	0.007	0.25	0.001	36
61	0.042	0.04	0.04	0.021	0.006	0.25	0.001	35
62	0.042	0.04	0.04	0.021	0.006	0.25	0.001	35
63	0.023	0.022	0.027	0.036	0.007	0.25	0.001	36
64	0.023	0.022	0.027	0.036	0.007	0.25	0.001	36
65	0.042	0.04	0.04	0.021	0.006	0.25	0.001	35
66	0.042	0.04	0.04	0.021	0.006	0.25	0.001	35
67	0.042	0.04	0.04	0.021	0.006	0.25	0.001	35
68	0.042	0.04	0.04	0.021	0.006	0.25	0.001	35
69	0.04	0.038	0.037	0.022	0.005	0.25	0.002	36
70	0.042	0.04	0.05	0.034	0.008	0.21	0.002	32
71	0.042	0.04	0.05	0.034	0.008	0.21	0.002	32
72	0.042	0.04	0.05	0.034	0.008	0.21	0.002	32
73	0.032	0.03	0.038	0.031	0.01	0.24	0.001	34
74	0.032	0.03	0.038	0.031	0.01	0.24	0.001	34
75	0.027	0.025	0.045	0.024	0.012	0.22	0.002	36
76	0.027	0.025	0.045	0.024	0.012	0.22	0.002	36
77	0.027	0.025	0.045	0.024	0.012	0.22	0.002	36
78	0.027	0.025	0.045	0.024	0.012	0.22	0.002	36
79	0.042	0.04	0.05	0.034	0.008	0.21	0.002	32
80	0.04	0.038	0.045	0.02	0.007	0.23	0.002	47
81	0.04	0.038	0.045	0.02	0.007	0.23	0.002	47
82	0.046	0.044	0.045	0.021	0.008	0.22	0.001	56
83	0.046	0.044	0.045	0.021	0.008	0.22	0.001	56
84	0.027	0.025	0.045	0.024	0.012	0.22	0.002	36
85	0.046	0.044	0.045	0.021	0.008	0.22	0.001	56
86	0.048	0.046	0.02	0.029	0.005	0.2	0.001	29
87	0.048	0.046	0.02	0.029	0.005	0.2	0.001	29
88	0.048	0.046	0.02	0.029	0.005	0.2	0.001	29
89	0.052	0.05	0.03	0.034	0.005	0.19	0.001	50
90	0.033	0.031	0.045	0.024	0.006	0.25	0.001	40
91	0.052	0.05	0.05	0.03	0.006	0.31	0.001	42
92	0.052	0.05	0.05	0.03	0.006	0.31	0.001	42
93	0.045	0.043	0.045	0.027	0.006	0.3	0.001	34
94	0.052	0.05	0.05	0.03	0.006	0.31	0.001	42
95	0.052	0.05	0.05	0.03	0.006	0.31	0.001	42
96	0.045	0.043	0.045	0.027	0.006	0.3	0.001	34
97	0.045	0.043	0.045	0.027	0.006	0.3	0.001	34
98	0.045	0.043	0.045	0.027	0.006	0.3	0.001	34
99	0.058	0.056	0.05	0.02	0.006	0.26	0.001	33

100	0.04	0.038	0.045	0.025	0.007	0.3	0.002	36
101	0.048	0.046	0.05	0.023	0.007	0.26	0.001	37
102	0.05	0.048	0.05	0.038	0.009	0.26	0.001	36
103	0.044	0.042	0.045	0.026	0.006	0.22	0.001	36
104	0.048	0.046	0.05	0.023	0.007	0.26	0.001	37
105	0.05	0.048	0.05	0.038	0.009	0.26	0.001	36
106	0.05	0.048	0.05	0.038	0.009	0.26	0.001	36
107	0.05	0.048	0.05	0.038	0.009	0.26	0.001	36
108	0.04	0.038	0.05	0.027	0.005	0.25	0.002	35
109	0.04	0.038	0.05	0.027	0.005	0.25	0.002	35
110	0.04	0.038	0.05	0.027	0.005	0.25	0.002	35
111	0.044	0.042	0.045	0.026	0.006	0.22	0.001	36
112	0.044	0.042	0.045	0.026	0.006	0.22	0.001	36
113	0.048	0.046	0.05	0.023	0.007	0.26	0.001	37
114	0.046	0.044	0.035	0.027	0.006	0.25	0.001	33
115	0.064	0.062	0.05	0.022	0.005	0.26	0.001	36
116	0.064	0.062	0.05	0.022	0.005	0.26	0.001	36
117	0.052	0.05	0.048	0.034	0.007	0.29	0.001	36
118	0.042	0.04	0.04	0.032	0.005	0.23	0.002	30
119	0.046	0.044	0.035	0.027	0.006	0.25	0.001	33
120	0.039	0.037	0.028	0.037	0.005	0.2	0.001	38
121	0.039	0.037	0.028	0.037	0.005	0.2	0.001	38
122	0.052	0.05	0.048	0.034	0.007	0.29	0.001	36
123	0.052	0.05	0.048	0.034	0.007	0.29	0.001	36
124	0.043	0.042	0.05	0.022	0.005	0.27	0.003	38
125	0.043	0.042	0.05	0.022	0.005	0.27	0.003	38
126	0.043	0.042	0.05	0.022	0.005	0.27	0.003	38
127	0.049	0.047	0.035	0.029	0.007	0.18	0.001	33
128	0.034	0.032	0.05	0.032	0.006	0.26	0.001	37
129	0.034	0.032	0.05	0.032	0.006	0.26	0.001	37
130	0.034	0.032	0.05	0.032	0.006	0.26	0.001	37
131	0.048	0.046	0.048	0.027	0.005	0.26	0.002	34
132	0.043	0.042	0.05	0.022	0.005	0.27	0.003	38
133	0.043	0.042	0.05	0.022	0.005	0.27	0.003	38
134	0.048	0.046	0.048	0.027	0.005	0.26	0.002	34
135	0.048	0.046	0.048	0.027	0.005	0.26	0.002	34
136	0.048	0.046	0.048	0.027	0.005	0.26	0.002	34
137	0.042	0.04	0.04	0.032	0.005	0.23	0.002	30
138	0.038	0.036	0.024	0.023	0.005	0.17	0.001	38
139	0.042	0.04	0.04	0.032	0.005	0.23	0.002	30
140	0.064	0.062	0.05	0.022	0.005	0.26	0.001	36
141	0.046	0.044	0.035	0.027	0.006	0.25	0.001	33
142	0.042	0.04	0.04	0.032	0.005	0.23	0.002	30
143	0.064	0.062	0.05	0.022	0.005	0.26	0.001	36
144	0.038	0.036	0.024	0.023	0.005	0.17	0.001	38
145	0.064	0.062	0.05	0.022	0.005	0.26	0.001	36
146	0.064	0.062	0.05	0.022	0.005	0.26	0.001	36
147	0.052	0.05	0.048	0.034	0.007	0.29	0.001	36
148	0.052	0.05	0.048	0.034	0.007	0.29	0.001	36
149	0.048	0.046	0.048	0.027	0.005	0.26	0.002	34

150	0.033	0.032	0.06	0.03	0.007	0.33	0.001	44
151	0.05	0.048	0.05	0.038	0.009	0.26	0.001	36
152	0.04	0.038	0.05	0.027	0.005	0.25	0.002	35
153	0.04	0.038	0.05	0.027	0.005	0.25	0.002	35
154	0.034	0.032	0.06	0.022	0.006	0.31	0.001	48
155	0.044	0.042	0.035	0.03	0.009	0.2	0.002	32
156	0.04	0.038	0.05	0.023	0.006	0.26	0.001	49
157	0.04	0.038	0.05	0.023	0.006	0.26	0.001	49
158	0.04	0.038	0.05	0.023	0.006	0.26	0.001	49
159	0.048	0.046	0.03	0.02	0.005	0.2	0.001	32
160	0.045	0.044	0.035	0.03	0.007	0.22	0.001	40
161	0.055	0.053	0.035	0.029	0.005	0.18	0.001	31
162	0.055	0.053	0.035	0.029	0.005	0.18	0.001	31
163	0.055	0.053	0.035	0.029	0.005	0.18	0.001	31
164	0.055	0.053	0.035	0.029	0.005	0.18	0.001	31
165	0.042	0.04	0.025	0.023	0.008	0.19	0.002	39
166	0.042	0.04	0.025	0.023	0.008	0.19	0.002	39
167	0.042	0.04	0.025	0.023	0.008	0.19	0.002	39
168	0.037	0.035	0.03	0.034	0.007	0.22	0.002	38
169	0.037	0.035	0.03	0.034	0.007	0.22	0.002	38
170	0.042	0.04	0.025	0.023	0.008	0.19	0.002	39
171	0.036	0.034	0.03	0.019	0.006	0.17	0.002	38
172	0.036	0.034	0.03	0.019	0.006	0.17	0.002	38
173	0.033	0.031	0.04	0.028	0.006	0.3	0.002	30
174	0.037	0.035	0.03	0.034	0.007	0.22	0.002	38
175	0.037	0.035	0.03	0.034	0.007	0.22	0.002	38
176	0.042	0.04	0.025	0.023	0.008	0.19	0.002	39
177	0.052	0.05	0.05	0.032	0.007	0.34	0.002	37
178	0.028	0.026	0.06	0.029	0.006	0.33	0.002	39
179	0.048	0.046	0.02	0.029	0.005	0.2	0.001	29
180	0.06	0.058	0.04	0.02	0.007	0.26	0.001	37
181	0.033	0.031	0.045	0.026	0.006	0.27	0.001	36
182	0.033	0.031	0.045	0.026	0.006	0.27	0.001	36
183	0.042	0.04	0.025	0.023	0.008	0.19	0.002	39
184	0.033	0.031	0.045	0.026	0.006	0.27	0.001	36
185	0.036	0.034	0.03	0.019	0.006	0.17	0.002	38
186	0.055	0.053	0.035	0.029	0.005	0.18	0.001	31
187	0.036	0.034	0.045	0.027	0.009	0.28	0.002	29
188	0.05	0.048	0.05	0.029	0.008	0.36	0.001	35
189	0.027	0.025	0.04	0.026	0.005	0.2	0.001	32
190	0.043	0.041	0.03	0.026	0.005	0.17	0.001	36
191	0.043	0.041	0.03	0.026	0.005	0.17	0.001	36
192	0.027	0.025	0.04	0.026	0.005	0.2	0.001	32
193	0.027	0.025	0.04	0.026	0.005	0.2	0.001	32
194	0.043	0.041	0.03	0.026	0.005	0.17	0.001	36
195	0.04	0.038	0.035	0.022	0.006	0.18	0.001	30
196	0.04	0.038	0.035	0.022	0.006	0.18	0.001	30
197	0.04	0.038	0.035	0.022	0.006	0.18	0.001	30
198	0.027	0.025	0.04	0.026	0.005	0.2	0.001	32
199	0.04	0.038	0.035	0.022	0.006	0.18	0.001	30

200	0.027	0.025	0.04	0.026	0.005	0.2	0.001	32
201	0.024	0.022	0.033	0.022	0.007	0.27	0.001	35
202	0.04	0.038	0.035	0.022	0.006	0.18	0.001	30
203	0.024	0.022	0.033	0.022	0.007	0.27	0.001	35
204	0.024	0.022	0.033	0.022	0.007	0.27	0.001	35
205	0.028	0.026	0.05	0.034	0.008	0.3	0.001	30
206	0.021	0.02	0.046	0.021	0.01	0.29	0.001	35
207	0.046	0.044	0.049	0.026	0.007	0.31	0.001	40
208	0.046	0.044	0.049	0.026	0.007	0.31	0.001	40
209	0.04	0.038	0.025	0.024	0.005	0.2	0.001	29
210	0.038	0.036	0.021	0.024	0.005	0.2	0.001	31
211	0.038	0.036	0.021	0.024	0.005	0.2	0.001	31
212	0.04	0.038	0.025	0.024	0.005	0.2	0.001	29
213	0.038	0.036	0.021	0.024	0.005	0.2	0.001	31
214	0.038	0.036	0.021	0.024	0.005	0.2	0.001	31
215	0.033	0.031	0.04	0.039	0.01	0.28	0.002	38
216	0.033	0.031	0.04	0.039	0.01	0.28	0.002	38
217	0.024	0.022	0.033	0.022	0.007	0.27	0.001	35
218	0.024	0.022	0.033	0.022	0.007	0.27	0.001	35
219	0.024	0.022	0.033	0.022	0.007	0.27	0.001	35
220	0.037	0.035	0.05	0.019	0.006	0.31	0.002	40
221	0.025	0.023	0.05	0.024	0.006	0.29	0.002	36
222	0.038	0.036	0.05	0.027	0.006	0.28	0.002	34
223	0.038	0.036	0.05	0.027	0.006	0.28	0.002	34
224	0.038	0.036	0.05	0.027	0.006	0.28	0.002	34
225	0.038	0.036	0.05	0.027	0.006	0.28	0.002	34
226	0.039	0.037	0.03	0.023	0.007	0.2	0.001	26
227	0.039	0.037	0.03	0.023	0.007	0.2	0.001	26
228	0.044	0.042	0.04	0.037	0.005	0.24	0.001	35
229	0.044	0.042	0.04	0.037	0.005	0.24	0.001	35
230	0.044	0.042	0.04	0.037	0.005	0.24	0.001	35
231	0.044	0.042	0.04	0.037	0.005	0.24	0.001	35
232	0.044	0.042	0.04	0.037	0.005	0.24	0.001	35
233	0.044	0.042	0.04	0.037	0.005	0.24	0.001	35
234	0.04	0.038	0.03	0.021	0.005	0.19	0.001	34
235	0.044	0.042	0.04	0.037	0.005	0.24	0.001	35
236	0.047	0.045	0.024	0.017	0.005	0.17	0.001	50
237	0.042	0.04	0.045	0.029	0.006	0.31	0.002	38
238	0.042	0.04	0.045	0.029	0.006	0.31	0.002	38
239	0.042	0.04	0.045	0.029	0.006	0.31	0.002	38
240	0.043	0.041	0.05	0.026	0.006	0.28	0.001	37
241	0.042	0.04	0.045	0.029	0.006	0.31	0.002	38
242	0.042	0.04	0.045	0.029	0.006	0.31	0.002	38
243	0.042	0.04	0.045	0.029	0.006	0.31	0.002	38
244	0.042	0.04	0.045	0.029	0.006	0.31	0.002	38
245	0.038	0.036	0.05	0.027	0.006	0.28	0.002	34
246	0.042	0.04	0.045	0.029	0.006	0.31	0.002	38
247	0.043	0.041	0.05	0.026	0.006	0.28	0.001	37
248	0.043	0.041	0.05	0.026	0.006	0.28	0.001	37
249	0.043	0.041	0.05	0.026	0.006	0.28	0.001	37

250	0.043	0.041	0.05	0.026	0.006	0.28	0.001	37
251	0.043	0.041	0.05	0.026	0.006	0.28	0.001	37
252	0.038	0.036	0.05	0.022	0.006	0.29	0.002	36
253	0.036	0.034	0.05	0.025	0.006	0.3	0.002	35
254	0.036	0.034	0.05	0.025	0.006	0.3	0.002	35
255	0.036	0.034	0.05	0.025	0.006	0.3	0.002	35
256	0.036	0.034	0.05	0.025	0.006	0.3	0.002	35
257	0.038	0.036	0.05	0.022	0.006	0.29	0.002	36
258	0.05	0.048	0.05	0.029	0.006	0.34	0.002	44
259	0.05	0.048	0.05	0.029	0.006	0.34	0.002	44
260	0.05	0.048	0.05	0.029	0.006	0.34	0.002	44
261	0.05	0.048	0.05	0.029	0.006	0.34	0.002	44
262	0.038	0.036	0.05	0.022	0.006	0.29	0.002	36
263	0.05	0.048	0.05	0.029	0.006	0.34	0.002	44
264	0.038	0.036	0.05	0.022	0.006	0.29	0.002	36
265	0.047	0.045	0.05	0.023	0.007	0.29	0.001	36
266	0.042	0.04	0.03	0.022	0.01	0.18	0.001	44
267	0.047	0.045	0.05	0.023	0.007	0.29	0.001	36
268	0.047	0.045	0.05	0.023	0.007	0.29	0.001	36
269	0.047	0.045	0.05	0.023	0.007	0.29	0.001	36
270	0.04	0.038	0.046	0.016	0.006	0.28	0.002	40
271	0.04	0.038	0.046	0.016	0.006	0.28	0.002	40
272	0.04	0.038	0.046	0.016	0.006	0.28	0.002	40
273	0.047	0.045	0.05	0.023	0.007	0.29	0.001	36
274	0.04	0.038	0.046	0.016	0.006	0.28	0.002	40
275	0.04	0.038	0.046	0.016	0.006	0.28	0.002	40
276	0.029	0.027	0.05	0.025	0.006	0.3	0.001	33
277	0.06	0.058	0.05	0.021	0.005	0.29	0.001	36
278	0.047	0.045	0.05	0.023	0.007	0.29	0.001	36
279	0.06	0.058	0.05	0.021	0.005	0.29	0.001	36
280	0.06	0.058	0.05	0.021	0.005	0.29	0.001	36
281	0.06	0.058	0.05	0.021	0.005	0.29	0.001	36
282	0.06	0.058	0.05	0.021	0.005	0.29	0.001	36
283	0.06	0.058	0.05	0.021	0.005	0.29	0.001	36
284	0.06	0.058	0.05	0.021	0.005	0.29	0.001	36
285	0.028	0.026	0.06	0.029	0.006	0.33	0.002	39
286	0.05	0.048	0.03	0.025	0.005	0.2	0.001	34
287	0.029	0.027	0.05	0.025	0.006	0.3	0.001	33
288	0.04	0.038	0.046	0.016	0.006	0.28	0.002	40
289	0.029	0.027	0.05	0.025	0.006	0.3	0.001	33
290	0.029	0.027	0.05	0.025	0.006	0.3	0.001	33
291	0.029	0.027	0.05	0.025	0.006	0.3	0.001	33
292	0.054	0.053	0.025	0.022	0.006	0.17	0.001	38
293	0.036	0.034	0.05	0.025	0.006	0.3	0.002	35
294	0.045	0.043	0.045	0.03	0.006	0.26	0.001	37
295	0.038	0.037	0.05	0.028	0.007	0.29	0.001	45
296	0.045	0.043	0.045	0.03	0.006	0.26	0.001	37
297	0.038	0.037	0.05	0.028	0.007	0.29	0.001	45
298	0.045	0.043	0.045	0.03	0.006	0.26	0.001	37
299	0.045	0.043	0.045	0.03	0.006	0.26	0.001	37

300	0.045	0.043	0.045	0.03	0.006	0.26	0.001	37
301	0.043	0.041	0.047	0.028	0.006	0.27	0.001	37
302	0.043	0.041	0.049	0.028	0.006	0.27	0.001	37
303	0.04	0.038	0.025	0.023	0.006	0.17	0.001	30
304	0.04	0.038	0.025	0.023	0.006	0.17	0.001	30
305	0.04	0.038	0.025	0.023	0.006	0.17	0.001	30
306	0.055	0.053	0.03	0.022	0.006	0.17	0.001	34
307	0.043	0.042	0.028	0.024	0.005	0.2	0.001	29
308	0.054	0.052	0.06	0.018	0.005	0.38	0.001	50
309	0.058	0.056	0.027	0.027	0.006	0.18	0.001	31
310	0.046	0.044	0.025	0.024	0.005	0.18	0.001	29
311	0.058	0.056	0.027	0.027	0.006	0.18	0.001	31
312	0.04	0.038	0.025	0.023	0.006	0.17	0.001	30
313	0.04	0.038	0.025	0.023	0.006	0.17	0.001	30
314	0.043	0.041	0.047	0.028	0.006	0.27	0.001	37
315	0.043	0.041	0.047	0.028	0.006	0.27	0.001	37
316	0.043	0.041	0.045	0.03	0.006	0.31	0.002	36
317	0.043	0.041	0.045	0.03	0.006	0.31	0.002	36
318	0.047	0.045	0.025	0.018	0.005	0.24	0.001	34
319	0.047	0.045	0.025	0.018	0.005	0.24	0.001	34
320	0.047	0.045	0.025	0.018	0.005	0.24	0.001	34
321	0.043	0.041	0.025	0.023	0.005	0.17	0.001	30
322	0.04	0.038	0.025	0.024	0.005	0.2	0.001	29
323	0.04	0.038	0.025	0.024	0.005	0.2	0.001	29
324	0.047	0.045	0.025	0.018	0.005	0.24	0.001	34
325	0.048	0.046	0.03	0.022	0.006	0.19	0.001	32
326	0.054	0.052	0.04	0.024	0.005	0.33	0.002	29
327	0.058	0.056	0.027	0.027	0.006	0.18	0.001	31
328	0.048	0.046	0.03	0.022	0.006	0.19	0.001	32
329	0.034	0.032	0.04	0.025	0.005	0.26	0.002	44
330	0.034	0.032	0.04	0.025	0.005	0.26	0.002	44
331	0.034	0.032	0.04	0.025	0.005	0.26	0.002	44
332	0.034	0.032	0.04	0.025	0.005	0.26	0.002	44
333	0.034	0.032	0.04	0.025	0.005	0.26	0.002	44
334	0.035	0.033	0.05	0.024	0.006	0.26	0.001	44
335	0.058	0.056	0.04	0.017	0.005	0.29	0.001	48
336	0.058	0.056	0.04	0.017	0.005	0.29	0.001	48
337	0.038	0.036	0.04	0.02	0.006	0.29	0.002	46
338	0.054	0.052	0.04	0.024	0.005	0.33	0.002	29
339	0.054	0.052	0.04	0.024	0.005	0.33	0.002	29
340	0.054	0.052	0.04	0.024	0.005	0.33	0.002	29
341	0.038	0.036	0.04	0.02	0.006	0.29	0.002	46
342	0.048	0.046	0.03	0.022	0.006	0.19	0.001	32
343	0.038	0.036	0.04	0.02	0.006	0.29	0.002	46
344	0.038	0.036	0.04	0.02	0.006	0.29	0.002	46
345	0.056	0.054	0.05	0.022	0.005	0.31	0.002	32
346	0.058	0.056	0.04	0.017	0.005	0.29	0.001	48
347	0.056	0.054	0.05	0.022	0.005	0.31	0.002	32
348	0.056	0.054	0.05	0.022	0.005	0.31	0.002	32
349	0.035	0.033	0.05	0.024	0.006	0.26	0.001	44

350	0.035	0.033	0.05	0.024	0.006	0.26	0.001	44
351	0.034	0.032	0.04	0.025	0.005	0.26	0.002	44
352	0.052	0.05	0.05	0.027	0.006	0.27	0.001	36
353	0.052	0.05	0.05	0.027	0.006	0.27	0.001	36
354	0.052	0.05	0.05	0.027	0.006	0.27	0.001	36
355	0.053	0.051	0.05	0.022	0.006	0.31	0.001	37
356	0.053	0.051	0.05	0.022	0.006	0.31	0.001	37
357	0.053	0.051	0.05	0.022	0.006	0.31	0.001	37
358	0.056	0.054	0.05	0.022	0.005	0.31	0.002	32
359	0.056	0.054	0.05	0.022	0.005	0.31	0.002	32
360	0.056	0.054	0.05	0.022	0.005	0.31	0.002	32
361	0.048	0.046	0.03	0.022	0.006	0.19	0.001	32
362	0.048	0.046	0.03	0.022	0.006	0.19	0.001	32
363	0.052	0.05	0.05	0.027	0.006	0.27	0.001	36
364	0.053	0.051	0.05	0.022	0.006	0.31	0.001	37
365	0.053	0.051	0.05	0.022	0.006	0.31	0.001	37
366	0.046	0.044	0.04	0.027	0.005	0.28	0.001	33
367	0.046	0.044	0.04	0.027	0.005	0.28	0.001	33
368	0.043	0.041	0.045	0.03	0.006	0.31	0.002	36
369	0.035	0.034	0.05	0.02	0.007	0.3	0.001	40
370	0.032	0.03	0.05	0.026	0.006	0.3	0.001	38
371	0.032	0.03	0.05	0.026	0.006	0.3	0.001	38
372	0.032	0.03	0.05	0.026	0.006	0.3	0.001	38
373	0.032	0.03	0.05	0.026	0.006	0.3	0.001	38
374	0.032	0.03	0.05	0.026	0.006	0.3	0.001	38
375	0.032	0.03	0.05	0.026	0.006	0.3	0.001	38
376	0.032	0.03	0.05	0.026	0.006	0.3	0.001	38
377	0.035	0.034	0.05	0.02	0.007	0.3	0.001	40
378	0.035	0.034	0.05	0.02	0.007	0.3	0.001	40
379	0.035	0.034	0.05	0.02	0.007	0.3	0.001	40
380	0.045	0.043	0.035	0.024	0.006	0.22	0.002	35
381	0.037	0.037	0.05	0.024	0.006	0.29	0.001	39
382	0.033	0.032	0.045	0.028	0.006	0.31	0.001	37
383	0.033	0.032	0.045	0.028	0.006	0.31	0.001	37
384	0.033	0.032	0.045	0.028	0.006	0.31	0.001	37
385	0.045	0.043	0.035	0.024	0.006	0.22	0.002	35
386	0.03	0.029	0.05	0.032	0.006	0.33	0.001	38
387	0.065	0.063	0.05	0.028	0.005	0.27	0.002	27
388	0.065	0.063	0.05	0.028	0.005	0.27	0.002	27
389	0.033	0.031	0.04	0.024	0.005	0.23	0.002	32
390	0.033	0.031	0.04	0.024	0.005	0.23	0.002	32
391	0.033	0.031	0.04	0.024	0.005	0.23	0.002	32
392	0.049	0.047	0.04	0.026	0.005	0.28	0.002	33
393	0.04	0.038	0.04	0.029	0.005	0.3	0.002	27
394	0.032	0.03	0.05	0.027	0.005	0.31	0.002	29
395	0.055	0.053	0.05	0.027	0.006	0.32	0.002	34
396	0.055	0.053	0.05	0.027	0.006	0.32	0.002	34
397	0.055	0.053	0.05	0.027	0.006	0.32	0.002	34
398	0.055	0.053	0.05	0.027	0.006	0.32	0.002	34
399	0.046	0.044	0.05	0.028	0.005	0.28	0.002	26

400	0.046	0.044	0.05	0.028	0.005	0.28	0.002	26
401	0.055	0.053	0.05	0.027	0.006	0.32	0.002	34
402	0.065	0.063	0.05	0.028	0.005	0.27	0.002	27
403	0.046	0.044	0.05	0.028	0.005	0.28	0.002	26
404	0.055	0.053	0.05	0.027	0.006	0.32	0.002	34
405	0.046	0.044	0.05	0.028	0.005	0.28	0.002	26
406	0.034	0.032	0.04	0.018	0.006	0.24	0.002	32
407	0.034	0.032	0.04	0.018	0.006	0.24	0.002	32
408	0.034	0.032	0.04	0.018	0.006	0.24	0.002	32
409	0.034	0.032	0.04	0.018	0.006	0.24	0.002	32
410	0.048	0.046	0.045	0.025	0.006	0.29	0.002	36
411	0.034	0.032	0.04	0.018	0.006	0.24	0.002	32
412	0.034	0.032	0.04	0.018	0.006	0.24	0.002	32
413	0.048	0.046	0.045	0.025	0.006	0.29	0.002	36
414	0.048	0.046	0.045	0.025	0.006	0.29	0.002	36
415	0.048	0.046	0.045	0.025	0.006	0.29	0.002	36
416	0.05	0.048	0.03	0.025	0.005	0.2	0.001	34
417	0.032	0.03	0.05	0.027	0.005	0.31	0.002	29
418	0.039	0.037	0.05	0.029	0.005	0.31	0.002	36
419	0.039	0.037	0.05	0.029	0.005	0.31	0.002	36
420	0.039	0.037	0.05	0.029	0.005	0.31	0.002	36
421	0.039	0.037	0.05	0.029	0.005	0.31	0.002	36
422	0.039	0.037	0.05	0.029	0.005	0.31	0.002	36
423	0.035	0.033	0.045	0.022	0.005	0.27	0.002	31
424	0.035	0.033	0.045	0.022	0.005	0.27	0.002	31
425	0.035	0.033	0.045	0.022	0.005	0.27	0.002	31
426	0.035	0.033	0.045	0.022	0.005	0.27	0.002	31
427	0.035	0.033	0.045	0.022	0.005	0.27	0.002	31
428	0.032	0.03	0.05	0.027	0.005	0.31	0.002	29
429	0.038	0.036	0.04	0.02	0.006	0.29	0.002	46
430	0.037	0.037	0.05	0.024	0.006	0.29	0.001	39
431	0.043	0.042	0.045	0.029	0.006	0.34	0.001	36
432	0.043	0.042	0.045	0.029	0.006	0.34	0.001	36
433	0.043	0.042	0.045	0.029	0.006	0.34	0.001	36
434	0.048	0.047	0.05	0.03	0.007	0.3	0.001	35
435	0.043	0.042	0.045	0.029	0.006	0.34	0.001	36

Nb	Ni	P	S	Si	Ti	V	FRT	CTT
0.001	0.01	0.016	0.018	0.008	0.001	0.001	864.92	650
0.002	0.012	0.01	0.01	0.014	0.002	0.002	867.39	570
0.001	0.011	0.013	0.015	0.01	0.001	0.001	876.93	570
0.001	0.011	0.016	0.013	0.007	0.001	0.001	891.54	650
0.001	0.015	0.018	0.01	0.011	0	0.001	870.86	570
0.001	0.012	0.009	0.014	0.009	0.001	0.001	862.47	570
0.001	0.012	0.02	0.019	0.014	0.002	0.001	882.26	570
0.001	0.01	0.014	0.015	0.008	0.001	0.001	867.95	570
0.001	0.01	0.014	0.011	0.012	0	0.002	868.88	570
0.001	0.011	0.019	0.019	0.011	0	0.002	864.91	570
0.001	0.011	0.019	0.019	0.011	0	0.002	866.08	570
0.001	0.011	0.01	0.02	0.008	0	0.002	860.76	570
0.001	0.011	0.01	0.02	0.008	0	0.002	877.05	570
0.001	0.011	0.01	0.02	0.008	0	0.002	871.6	570
0.001	0.01	0.014	0.011	0.012	0	0.002	879.62	570
0.001	0.01	0.014	0.011	0.012	0	0.002	877.82	570
0.001	0.01	0.014	0.011	0.012	0	0.002	883.87	570
0.001	0.011	0.019	0.019	0.011	0	0.002	883.75	570
0.001	0.011	0.019	0.019	0.011	0	0.002	882.21	570
0.001	0.011	0.019	0.019	0.011	0	0.002	890.62	570
0.002	0.01	0.015	0.013	0.006	0.002	0.002	861.61	570
0.002	0.01	0.015	0.013	0.006	0.002	0.002	859.41	570
0.001	0.011	0.016	0.013	0.008	0	0.001	884.59	570
0.001	0.011	0.014	0.011	0.01	0.001	0.001	875	570
0.001	0.009	0.01	0.02	0.005	0.001	0.001	896.41	570
0.001	0.011	0.014	0.012	0.006	0	0.002	870.33	650
0.002	0.012	0.013	0.009	0.01	0.002	0.002	890.15	570
0.001	0.011	0.012	0.01	0.01	0	0.001	877.03	570
0.001	0.01	0.008	0.017	0.007	0.001	0.002	862.89	570
0.001	0.01	0.008	0.017	0.007	0.001	0.002	855.39	570
0.001	0.01	0.008	0.017	0.007	0.001	0.002	865.61	570
0.001	0.01	0.02	0.015	0.007	0.001	0.001	862.86	570
0.001	0.01	0.013	0.015	0.017	0	0.001	869.79	570
0.001	0.01	0.013	0.015	0.017	0	0.001	871.43	570
0.002	0.01	0.01	0.01	0.007	0.002	0.002	867.05	570
0.002	0.01	0.01	0.01	0.007	0.002	0.002	875.54	570
0.002	0.01	0.01	0.01	0.007	0.002	0.002	883.65	570
0.002	0.01	0.01	0.01	0.007	0.002	0.002	874.14	570
0.001	0.01	0.013	0.01	0.017	0.002	0.001	878.76	570
0.001	0.01	0.013	0.01	0.017	0.002	0.001	887.4	570
0.001	0.01	0.013	0.014	0.008	0.001	0.001	869.85	570
0.001	0.01	0.013	0.014	0.008	0.001	0.001	875.11	570
0.001	0.009	0.013	0.01	0.013	0.002	0.001	861.3	570
0.001	0.01	0.01	0.014	0.01	0	0.001	881.82	570
0.002	0.011	0.009	0.007	0.013	0.002	0.002	880.06	570
0.001	0.009	0.009	0.01	0.009	0	0.001	874.74	570
0.001	0.01	0.009	0.013	0.011	0.001	0.001	859.65	570
0.001	0.009	0.009	0.01	0.009	0	0.001	872.24	570
0.002	0.011	0.009	0.007	0.013	0.002	0.002	858.95	570

0.001	0.01	0.01	0.009	0.009	0	0.001	868.51	570
0.001	0.011	0.012	0.01	0.01	0.002	0.001	868.87	570
0.001	0.01	0.013	0.009	0.011	0.001	0.001	866.8	570
0.002	0.01	0.012	0.009	0.01	0.002	0.002	872.41	570
0.001	0.01	0.015	0.02	0.004	0	0.002	863.92	570
0.001	0.01	0.013	0.006	0.028	0	0.002	885.43	570
0.001	0.01	0.013	0.012	0.009	0.001	0.001	874.83	570
0.001	0.01	0.01	0.018	0.008	0.001	0.001	865.76	570
0.001	0.009	0.014	0.009	0.018	0.001	0.001	883.66	570
0.001	0.009	0.014	0.009	0.018	0.001	0.001	883.86	570
0.001	0.011	0.02	0.013	0.008	0.001	0.001	876.24	570
0.001	0.011	0.01	0.014	0.009	0.001	0.001	873.81	570
0.001	0.011	0.01	0.014	0.009	0.001	0.001	886.77	570
0.001	0.011	0.02	0.013	0.008	0.001	0.001	880.61	570
0.001	0.011	0.02	0.013	0.008	0.001	0.001	873.02	570
0.001	0.011	0.01	0.014	0.009	0.001	0.001	878.06	570
0.001	0.011	0.01	0.014	0.009	0.001	0.001	870.62	570
0.001	0.011	0.01	0.014	0.009	0.001	0.001	879.66	570
0.001	0.011	0.01	0.014	0.009	0.001	0.001	893.57	570
0.001	0.01	0.01	0.009	0.009	0	0.001	868.25	570
0.002	0.012	0.01	0.007	0.014	0.002	0.002	872.33	570
0.002	0.012	0.01	0.007	0.014	0.002	0.002	866.9	570
0.002	0.012	0.01	0.007	0.014	0.002	0.002	873.72	570
0.001	0.012	0.018	0.013	0.01	0	0.001	868.62	570
0.001	0.012	0.018	0.013	0.01	0	0.001	877.12	570
0.002	0.013	0.01	0.007	0.012	0.002	0.002	872.45	570
0.002	0.013	0.01	0.007	0.012	0.002	0.002	876.74	570
0.002	0.013	0.01	0.007	0.012	0.002	0.002	874.28	570
0.002	0.013	0.01	0.007	0.012	0.002	0.002	880.12	570
0.002	0.012	0.01	0.007	0.014	0.002	0.002	888.44	570
0.002	0.011	0.008	0.009	0.013	0.002	0.002	872.48	570
0.002	0.011	0.008	0.009	0.013	0.002	0.002	873.55	570
0.001	0.01	0.007	0.01	0.012	0.001	0.001	864.48	570
0.001	0.01	0.007	0.01	0.012	0.001	0.001	872.08	570
0.002	0.013	0.01	0.007	0.012	0.002	0.002	869.01	570
0.001	0.01	0.007	0.01	0.012	0.001	0.001	870.78	570
0.001	0.009	0.019	0.012	0.007	0	0.001	871.59	570
0.001	0.009	0.019	0.012	0.007	0	0.001	870.92	570
0.001	0.009	0.019	0.012	0.007	0	0.001	869.58	570
0.001	0.009	0.012	0.003	0.027	0	0.001	872.27	570
0.001	0.011	0.015	0.006	0.018	0.001	0.001	878.6	570
0.001	0.01	0.02	0.007	0.01	0.001	0.001	869.61	570
0.001	0.01	0.02	0.007	0.01	0.001	0.001	881.79	570
0.001	0.012	0.01	0.012	0.008	0.001	0.001	872.02	570
0.001	0.01	0.02	0.007	0.01	0.001	0.001	879.44	570
0.001	0.01	0.02	0.007	0.01	0.001	0.001	877.84	570
0.001	0.012	0.01	0.012	0.008	0.001	0.001	873.32	570
0.001	0.012	0.01	0.012	0.008	0.001	0.001	879.47	570
0.001	0.012	0.01	0.012	0.008	0.001	0.001	881.7	570
0.001	0.009	0.009	0.012	0.01	0.001	0.001	875.46	570

0.002	0.01	0.01	0.01	0.016	0.002	0.002	881.5	570
0.001	0.012	0.011	0.008	0.018	0.001	0.001	871.68	570
0.001	0.014	0.011	0.01	0.01	0.001	0.001	891.88	570
0.001	0.01	0.01	0.01	0.01	0.001	0.001	880.7	570
0.001	0.012	0.011	0.008	0.018	0.001	0.001	890.8	570
0.001	0.014	0.011	0.01	0.01	0.001	0.001	874.8	570
0.001	0.014	0.011	0.01	0.01	0.001	0.001	888.39	570
0.001	0.014	0.011	0.01	0.01	0.001	0.001	867.8	570
0.001	0.01	0.016	0.011	0.008	0.002	0.001	884.02	570
0.001	0.01	0.016	0.011	0.008	0.002	0.001	874.04	570
0.001	0.01	0.016	0.011	0.008	0.002	0.001	880.24	570
0.001	0.01	0.01	0.01	0.01	0.001	0.001	875.71	570
0.001	0.01	0.01	0.01	0.01	0.001	0.001	879.14	570
0.001	0.012	0.011	0.008	0.018	0.001	0.001	870.32	570
0.001	0.01	0.013	0.014	0.01	0.001	0.001	871.08	570
0.001	0.009	0.01	0.014	0.008	0.001	0.001	878.76	570
0.001	0.009	0.01	0.014	0.008	0.001	0.001	870.28	570
0.001	0.015	0.01	0.01	0.012	0.001	0.001	876.71	570
0.001	0.016	0.015	0.013	0.011	0.001	0.002	880.05	570
0.001	0.01	0.013	0.014	0.01	0.001	0.001	888.78	570
0.001	0.014	0.012	0.01	0.011	0	0.001	891.81	570
0.001	0.014	0.012	0.01	0.011	0	0.001	878.81	570
0.001	0.015	0.01	0.01	0.012	0.001	0.001	894.21	570
0.001	0.015	0.01	0.01	0.012	0.001	0.001	850.23	570
0.001	0.01	0.009	0.014	0.008	0	0.001	869.62	570
0.001	0.01	0.009	0.014	0.008	0	0.001	872.22	570
0.001	0.01	0.009	0.014	0.008	0	0.001	865.61	570
0.001	0.01	0.01	0.008	0.015	0.001	0.001	864.34	570
0.001	0.011	0.012	0.007	0.01	0.001	0.001	866.5	570
0.001	0.011	0.012	0.007	0.01	0.001	0.001	874.65	570
0.001	0.011	0.012	0.007	0.01	0.001	0.001	871.05	570
0.001	0.01	0.013	0.01	0.008	0	0.001	866.28	570
0.001	0.01	0.009	0.014	0.008	0	0.001	865.79	570
0.001	0.01	0.009	0.014	0.008	0	0.001	862.39	570
0.001	0.01	0.013	0.01	0.008	0	0.001	865.12	570
0.001	0.01	0.013	0.01	0.008	0	0.001	874.78	570
0.001	0.01	0.013	0.01	0.008	0	0.001	858.58	570
0.001	0.016	0.015	0.013	0.011	0.001	0.002	871.8	570
0.001	0.01	0.01	0.009	0.006	0.001	0.001	867.34	570
0.001	0.016	0.015	0.013	0.011	0.001	0.002	880.27	570
0.001	0.009	0.01	0.014	0.008	0.001	0.001	866.32	570
0.001	0.01	0.013	0.014	0.01	0.001	0.001	877.07	570
0.001	0.016	0.015	0.013	0.011	0.001	0.002	866.97	570
0.001	0.009	0.01	0.014	0.008	0.001	0.001	870.87	570
0.001	0.01	0.01	0.009	0.006	0.001	0.001	868.49	570
0.001	0.009	0.01	0.014	0.008	0.001	0.001	870.33	570
0.001	0.009	0.01	0.014	0.008	0.001	0.001	867.02	570
0.001	0.015	0.01	0.01	0.012	0.001	0.001	874.76	570
0.001	0.015	0.01	0.01	0.012	0.001	0.001	873.3	570
0.001	0.01	0.013	0.01	0.008	0	0.001	875.45	570

0.001	0.011	0.013	0.018	0.011	0.001	0.001	871.46	570
0.001	0.014	0.011	0.01	0.01	0.001	0.001	865.44	570
0.001	0.01	0.016	0.011	0.008	0.002	0.001	860.02	570
0.001	0.01	0.016	0.011	0.008	0.002	0.001	868.57	570
0.001	0.01	0.016	0.012	0.01	0.001	0.001	861.29	570
0.001	0.01	0.016	0.01	0.012	0.002	0.001	882.45	570
0.001	0.01	0.008	0.011	0.008	0	0.001	865.43	570
0.001	0.01	0.008	0.011	0.008	0	0.001	873.86	570
0.001	0.01	0.008	0.011	0.008	0	0.001	866.1	570
0.001	0.01	0.011	0.01	0.009	0.001	0.001	877.39	570
0.001	0.012	0.015	0.013	0.012	0.001	0.001	891.92	570
0	0.009	0.013	0.01	0.008	0	0.001	874.54	570
0	0.009	0.013	0.01	0.008	0	0.001	880.59	570
0	0.009	0.013	0.01	0.008	0	0.001	880.15	570
0	0.009	0.013	0.01	0.008	0	0.001	882.3	570
0.001	0.012	0.008	0.014	0.013	0.002	0.001	871.97	570
0.001	0.012	0.008	0.014	0.013	0.002	0.001	874.94	570
0.001	0.012	0.008	0.014	0.013	0.002	0.001	877.73	570
0.002	0.01	0.016	0.014	0.009	0.002	0.001	877.31	570
0.002	0.01	0.016	0.014	0.009	0.002	0.001	882.08	570
0.001	0.012	0.008	0.014	0.013	0.002	0.001	866.46	570
0.001	0.009	0.008	0.01	0.007	0.002	0.001	879.96	570
0.001	0.009	0.008	0.01	0.007	0.002	0.001	878.81	570
0.001	0.01	0.012	0.015	0.019	0.002	0.001	879.48	570
0.002	0.01	0.016	0.014	0.009	0.002	0.001	878.93	570
0.002	0.01	0.016	0.014	0.009	0.002	0.001	880.95	570
0.001	0.012	0.008	0.014	0.013	0.002	0.001	880.53	570
0.001	0.009	0.013	0.015	0.008	0.002	0.001	889.51	570
0.001	0.009	0.024	0.019	0.013	0.002	0.001	893.19	570
0.001	0.009	0.019	0.012	0.007	0	0.001	894.41	570
0.001	0.01	0.008	0.015	0.008	0.001	0.001	883.13	570
0.001	0.009	0.01	0.018	0.007	0.001	0.001	878.08	570
0.001	0.009	0.01	0.018	0.007	0.001	0.001	887.52	570
0.001	0.012	0.008	0.014	0.013	0.002	0.001	875.9	570
0.001	0.009	0.01	0.018	0.007	0.001	0.001	880.26	570
0.001	0.009	0.008	0.01	0.007	0.002	0.001	878.34	570
0	0.009	0.013	0.01	0.008	0	0.001	877.81	570
0.002	0.009	0.01	0.014	0.009	0.002	0.001	875.96	570
0.001	0.009	0.008	0.01	0.009	0.001	0.001	877.81	570
0.003	0.009	0.017	0.01	0.015	0	0.001	876.63	570
0	0.01	0.015	0.01	0.011	0	0.001	881.68	570
0	0.01	0.015	0.01	0.011	0	0.001	876.29	570
0.003	0.009	0.017	0.01	0.015	0	0.001	875.73	570
0.003	0.009	0.017	0.01	0.015	0	0.001	873.68	570
0	0.01	0.015	0.01	0.011	0	0.001	878.93	570
0	0.009	0.012	0.008	0.011	0	0.001	870.8	570
0	0.009	0.012	0.008	0.011	0	0.001	888.95	570
0	0.009	0.012	0.008	0.011	0	0.001	887.89	570
0.003	0.009	0.017	0.01	0.015	0	0.001	890.2	570
0	0.009	0.012	0.008	0.011	0	0.001	886.07	570

0.003	0.009	0.017	0.01	0.015	0	0.001	892.39	570
0.001	0.01	0.015	0.01	0.012	0	0.001	889.83	570
0	0.009	0.012	0.008	0.011	0	0.001	890.89	570
0.001	0.01	0.015	0.01	0.012	0	0.001	885.83	570
0.001	0.01	0.015	0.01	0.012	0	0.001	877.49	570
0.004	0.01	0.017	0.011	0.008	0	0.001	901.14	570
0.004	0.01	0.018	0.017	0.01	0.003	0.002	896.73	570
0.001	0.008	0.015	0.008	0.01	0	0.001	877.03	570
0.001	0.008	0.015	0.008	0.01	0	0.001	876.76	570
0.001	0.01	0.009	0.014	0.008	0.001	0.001	881.91	570
0.001	0.01	0.01	0.014	0.008	0.001	0.001	896.61	570
0.001	0.01	0.01	0.014	0.008	0.001	0.001	883.36	570
0.001	0.01	0.009	0.014	0.008	0.001	0.001	897.03	570
0.001	0.01	0.01	0.014	0.008	0.001	0.001	880.94	570
0.001	0.01	0.01	0.014	0.008	0.001	0.001	889.18	570
0.001	0.01	0.011	0.012	0.008	0	0.002	891.11	570
0.001	0.01	0.011	0.012	0.008	0	0.002	880.16	570
0.001	0.01	0.015	0.01	0.012	0	0.001	866.24	570
0.001	0.01	0.015	0.01	0.012	0	0.001	868.37	570
0.001	0.01	0.015	0.01	0.012	0	0.001	868.24	570
0.001	0.008	0.009	0.01	0.008	0	0.002	869.65	570
0.001	0.01	0.014	0.01	0.014	0.002	0.001	885.12	570
0.001	0.01	0.011	0.01	0.01	0.002	0.001	886.25	570
0.001	0.01	0.011	0.01	0.01	0.002	0.001	878.86	570
0.001	0.01	0.011	0.01	0.01	0.002	0.001	885.8	570
0.001	0.01	0.011	0.01	0.01	0.002	0.001	879.06	570
0.001	0.008	0.01	0.01	0.008	0	0.001	873.25	570
0.001	0.008	0.01	0.01	0.008	0	0.001	886	570
0	0.01	0.018	0.013	0.01	0	0.001	876.48	570
0	0.01	0.018	0.013	0.01	0	0.001	878.65	570
0	0.01	0.018	0.013	0.01	0	0.001	882.49	570
0	0.01	0.018	0.013	0.01	0	0.001	887.91	570
0	0.01	0.018	0.013	0.01	0	0.001	878.23	570
0	0.01	0.018	0.013	0.01	0	0.001	888.74	570
0.001	0.01	0.01	0.01	0.009	0	0.001	874.78	570
0	0.01	0.018	0.013	0.01	0	0.001	893.56	570
0.001	0.01	0.007	0.01	0.009	0	0.001	868.18	570
0.001	0.011	0.016	0.015	0.01	0.002	0.001	892.51	570
0.001	0.011	0.016	0.015	0.01	0.002	0.001	889.45	570
0.001	0.011	0.016	0.015	0.01	0.002	0.001	882.68	570
0.001	0.01	0.014	0.014	0.009	0.001	0.002	884.32	570
0.001	0.011	0.016	0.015	0.01	0.002	0.001	880.91	570
0.001	0.011	0.016	0.015	0.01	0.002	0.001	888.41	570
0.001	0.011	0.016	0.015	0.01	0.002	0.001	879.54	570
0.001	0.011	0.016	0.015	0.01	0.002	0.001	868.14	570
0.001	0.01	0.011	0.01	0.01	0.002	0.001	879.36	570
0.001	0.011	0.016	0.015	0.01	0.002	0.001	887.58	570
0.001	0.01	0.014	0.014	0.009	0.001	0.002	879.71	570
0.001	0.01	0.014	0.014	0.009	0.001	0.002	884.42	570
0.001	0.01	0.014	0.014	0.009	0.001	0.002	886.23	570

0.001	0.01	0.014	0.014	0.009	0.001	0.002	884.11	570
0.001	0.01	0.014	0.014	0.009	0.001	0.002	886.34	570
0.001	0.009	0.015	0.011	0.011	0.002	0.001	880.76	570
0.001	0.009	0.012	0.01	0.01	0.002	0.001	888.41	570
0.001	0.009	0.012	0.01	0.01	0.002	0.001	875.97	570
0.001	0.009	0.012	0.01	0.01	0.002	0.001	890.03	570
0.001	0.009	0.012	0.01	0.01	0.002	0.001	881.6	570
0.001	0.009	0.015	0.011	0.011	0.002	0.001	888.97	570
0.001	0.01	0.014	0.011	0.014	0.002	0.001	871.08	570
0.001	0.01	0.014	0.011	0.014	0.002	0.001	884.87	570
0.001	0.01	0.014	0.011	0.014	0.002	0.001	883.41	570
0.001	0.01	0.014	0.011	0.014	0.002	0.001	885.32	570
0.001	0.009	0.015	0.011	0.011	0.002	0.001	882.42	570
0.001	0.01	0.014	0.011	0.014	0.002	0.001	882.81	570
0.001	0.009	0.015	0.011	0.011	0.002	0.001	889.17	570
0.001	0.01	0.01	0.015	0.01	0.001	0.001	871.55	570
0.002	0.011	0.01	0.01	0.01	0.001	0.001	868.85	570
0.001	0.01	0.01	0.015	0.01	0.001	0.001	872.81	570
0.001	0.01	0.01	0.015	0.01	0.001	0.001	868.73	570
0.001	0.01	0.01	0.015	0.01	0.001	0.001	880.1	570
0.001	0.011	0.009	0.014	0.01	0	0.001	871.55	570
0.001	0.011	0.009	0.014	0.01	0	0.001	883.03	570
0.001	0.011	0.009	0.014	0.01	0	0.001	872.44	570
0.001	0.01	0.01	0.015	0.01	0.001	0.001	878.84	570
0.001	0.011	0.009	0.014	0.01	0	0.001	872.65	570
0.001	0.011	0.009	0.014	0.01	0	0.001	878.89	570
0.001	0.01	0.023	0.013	0.01	0.001	0.001	882.08	570
0.001	0.01	0.011	0.015	0.009	0.001	0.001	884.4	570
0.001	0.01	0.01	0.015	0.01	0.001	0.001	881.27	570
0.001	0.01	0.011	0.015	0.009	0.001	0.001	885.53	570
0.001	0.01	0.011	0.015	0.009	0.001	0.001	879.66	570
0.001	0.01	0.011	0.015	0.009	0.001	0.001	887.61	570
0.001	0.01	0.011	0.015	0.009	0.001	0.001	886.03	570
0.001	0.01	0.011	0.015	0.009	0.001	0.001	892.8	570
0.001	0.01	0.011	0.015	0.009	0.001	0.001	885.48	570
0.001	0.009	0.024	0.019	0.013	0.002	0.001	871.28	570
0.001	0.01	0.013	0.01	0.011	0.001	0.001	858.19	570
0.001	0.01	0.023	0.013	0.01	0.001	0.001	865.81	570
0.001	0.011	0.009	0.014	0.01	0	0.001	865.11	570
0.001	0.01	0.023	0.013	0.01	0.001	0.001	865.04	570
0.001	0.01	0.023	0.013	0.01	0.001	0.001	869.89	570
0.001	0.01	0.023	0.013	0.01	0.001	0.001	864.45	570
0.001	0.011	0.011	0.009	0.009	0.001	0.001	868.22	570
0.001	0.009	0.012	0.01	0.01	0.002	0.001	870.27	570
0.001	0.01	0.017	0.012	0.009	0	0.001	886.16	570
0.001	0.011	0.014	0.007	0.014	0.001	0.001	869.78	570
0.001	0.01	0.017	0.012	0.009	0	0.001	873.39	570
0.001	0.011	0.014	0.007	0.014	0.001	0.001	878.78	570
0.001	0.01	0.017	0.012	0.009	0	0.001	873.08	570
0.001	0.01	0.017	0.012	0.009	0	0.001	877.7	570

0.001	0.01	0.017	0.012	0.009	0	0.001	878.3	570
0.001	0.014	0.011	0.011	0.008	0	0.001	883.96	570
0.001	0.014	0.011	0.011	0.008	0	0.001	873.05	570
0.001	0.01	0.01	0.014	0.007	0	0.001	873.69	570
0.001	0.01	0.01	0.014	0.007	0	0.001	881.65	570
0.001	0.01	0.01	0.014	0.007	0	0.001	875.96	570
0.001	0.01	0.01	0.01	0.01	0	0.001	883.6	570
0.001	0.01	0.013	0.012	0.007	0.001	0.001	877.08	570
0.001	0.009	0.009	0.014	0.011	0.001	0.001	887	570
0.001	0.011	0.01	0.01	0.008	0	0.001	877.15	570
0.001	0.01	0.012	0.01	0.012	0	0.001	877.09	570
0.001	0.011	0.01	0.01	0.008	0	0.001	876.76	570
0.001	0.01	0.01	0.014	0.007	0	0.001	875.81	570
0.001	0.01	0.01	0.014	0.007	0	0.001	872.69	570
0.001	0.014	0.011	0.011	0.008	0	0.001	890.12	570
0.001	0.014	0.011	0.011	0.008	0	0.001	887.72	570
0.001	0.01	0.013	0.014	0.011	0	0.002	889.48	570
0.001	0.01	0.013	0.014	0.011	0	0.002	890.07	570
0.001	0.01	0.009	0.016	0.005	0.001	0.001	871.28	570
0.001	0.01	0.009	0.016	0.005	0.001	0.001	867.61	570
0.001	0.01	0.009	0.016	0.005	0.001	0.001	870.71	570
0.001	0.01	0.01	0.01	0.009	0	0.001	872.18	570
0.001	0.01	0.009	0.014	0.008	0.001	0.001	869.22	570
0.001	0.01	0.009	0.014	0.008	0.001	0.001	868.34	570
0.001	0.01	0.009	0.016	0.005	0.001	0.001	866.61	570
0.001	0.009	0.012	0.015	0.007	0	0.001	866.97	570
0.001	0.012	0.011	0.011	0.01	0.002	0.001	876.36	570
0.001	0.011	0.01	0.01	0.008	0	0.001	873.81	570
0.001	0.009	0.012	0.015	0.007	0	0.001	866.02	570
0.001	0.018	0.016	0.017	0.008	0.002	0.001	879.92	570
0.001	0.018	0.016	0.017	0.008	0.002	0.001	882.2	570
0.001	0.018	0.016	0.017	0.008	0.002	0.001	889.45	570
0.001	0.018	0.016	0.017	0.008	0.002	0.001	879.67	570
0.001	0.018	0.016	0.017	0.008	0.002	0.001	889.32	570
0.001	0.01	0.012	0.02	0.01	0	0.001	883.79	570
0.001	0.01	0.011	0.016	0.008	0	0.001	894.47	570
0.001	0.01	0.011	0.016	0.008	0	0.001	882.57	570
0.001	0.009	0.011	0.015	0.01	0	0.001	878.72	570
0.001	0.012	0.011	0.011	0.01	0.002	0.001	883.91	570
0.001	0.012	0.011	0.011	0.01	0.002	0.001	878.58	570
0.001	0.012	0.011	0.011	0.01	0.002	0.001	881.41	570
0.001	0.009	0.011	0.015	0.01	0	0.001	869.75	570
0.001	0.009	0.012	0.015	0.007	0	0.001	865.16	570
0.001	0.009	0.011	0.015	0.01	0	0.001	870.77	570
0.001	0.009	0.011	0.015	0.01	0	0.001	878.69	570
0.001	0.009	0.011	0.014	0.009	0.002	0.001	867.24	570
0.001	0.01	0.011	0.016	0.008	0	0.001	874.28	570
0.001	0.009	0.011	0.014	0.009	0.002	0.001	881.92	570
0.001	0.009	0.011	0.014	0.009	0.002	0.001	883.45	570
0.001	0.01	0.012	0.02	0.01	0	0.001	882.33	570

0.001	0.01	0.012	0.02	0.01	0	0.001	870.25	570
0.001	0.018	0.016	0.017	0.008	0.002	0.001	884.44	570
0.001	0.009	0.012	0.012	0.012	0.001	0.001	881.97	570
0.001	0.009	0.012	0.012	0.012	0.001	0.001	885.96	570
0.001	0.009	0.012	0.012	0.012	0.001	0.001	887.14	570
0.001	0.009	0.011	0.015	0.008	0.001	0.001	886.34	570
0.001	0.009	0.011	0.015	0.008	0.001	0.001	879.25	570
0.001	0.009	0.011	0.015	0.008	0.001	0.001	886.88	570
0.001	0.009	0.011	0.014	0.009	0.002	0.001	872.97	570
0.001	0.009	0.011	0.014	0.009	0.002	0.001	882.68	570
0.001	0.009	0.011	0.014	0.009	0.002	0.001	874.22	570
0.001	0.009	0.012	0.015	0.007	0	0.001	877.21	570
0.001	0.009	0.012	0.015	0.007	0	0.001	871.54	570
0.001	0.009	0.012	0.012	0.012	0.001	0.001	885.33	570
0.001	0.009	0.011	0.015	0.008	0.001	0.001	878.17	570
0.001	0.009	0.011	0.015	0.008	0.001	0.001	883.03	570
0.001	0.01	0.012	0.007	0.009	0	0.001	888.82	570
0.001	0.01	0.012	0.007	0.009	0	0.001	882.68	570
0.001	0.01	0.013	0.014	0.011	0	0.002	883.86	570
0.001	0.016	0.011	0.012	0.008	0.002	0.001	890.58	570
0.001	0.009	0.012	0.015	0.01	0.001	0.001	893.88	570
0.001	0.009	0.012	0.015	0.01	0.001	0.001	884.23	570
0.001	0.009	0.012	0.015	0.01	0.001	0.001	879	570
0.001	0.009	0.012	0.015	0.01	0.001	0.001	869.5	570
0.001	0.009	0.012	0.015	0.01	0.001	0.001	876.36	570
0.001	0.009	0.012	0.015	0.01	0.001	0.001	872.02	570
0.001	0.009	0.012	0.015	0.01	0.001	0.001	874.56	570
0.001	0.016	0.011	0.012	0.008	0.002	0.001	885.89	570
0.001	0.016	0.011	0.012	0.008	0.002	0.001	877.84	570
0.001	0.016	0.011	0.012	0.008	0.002	0.001	884.08	570
0.001	0.01	0.011	0.009	0.013	0.002	0.001	873.8	570
0.001	0.014	0.013	0.015	0.007	0.001	0.001	881.95	570
0.001	0.01	0.018	0.012	0.008	0.001	0.001	883.11	570
0.001	0.01	0.018	0.012	0.008	0.001	0.001	879.1	570
0.001	0.01	0.018	0.012	0.008	0.001	0.001	885.25	570
0.001	0.01	0.011	0.009	0.013	0.002	0.001	870.88	570
0.001	0.009	0.017	0.012	0.01	0.001	0.001	875.71	570
0.002	0.01	0.015	0.013	0.006	0.002	0.002	870.01	570
0.002	0.01	0.015	0.013	0.006	0.002	0.002	880.82	570
0.001	0.011	0.01	0.02	0.008	0	0.002	880.2	570
0.001	0.011	0.01	0.02	0.008	0	0.002	873.52	570
0.001	0.011	0.01	0.02	0.008	0	0.002	876.1	570
0.001	0.01	0.014	0.011	0.012	0	0.002	876.28	570
0.001	0.01	0.016	0.02	0.008	0	0.002	884.84	570
0.002	0.01	0.016	0.018	0.012	0.002	0.002	872.87	570
0.001	0.01	0.013	0.016	0.009	0	0.002	890.06	570
0.001	0.01	0.013	0.016	0.009	0	0.002	879.91	570
0.001	0.01	0.013	0.016	0.009	0	0.002	880.93	570
0.001	0.01	0.013	0.016	0.009	0	0.002	879.48	570
0.001	0.01	0.015	0.016	0.009	0.002	0.001	877.78	570

0.001	0.01	0.015	0.016	0.009	0.002	0.001	871.36	570
0.001	0.01	0.013	0.016	0.009	0	0.002	884.34	570
0.002	0.01	0.015	0.013	0.006	0.002	0.002	876.15	570
0.001	0.01	0.015	0.016	0.009	0.002	0.001	876.64	570
0.001	0.01	0.013	0.016	0.009	0	0.002	882.89	570
0.001	0.01	0.015	0.016	0.009	0.002	0.001	870.26	570
0.001	0.01	0.015	0.014	0.011	0.002	0.001	878	570
0.001	0.01	0.015	0.014	0.011	0.002	0.001	876.97	570
0.001	0.01	0.015	0.014	0.011	0.002	0.001	878.2	570
0.001	0.01	0.015	0.014	0.011	0.002	0.001	877.17	570
0.001	0.012	0.013	0.016	0.009	0.002	0.001	875.17	570
0.001	0.01	0.015	0.014	0.011	0.002	0.001	877.96	570
0.001	0.01	0.015	0.014	0.011	0.002	0.001	876.58	570
0.001	0.012	0.013	0.016	0.009	0.002	0.001	871.71	570
0.001	0.012	0.013	0.016	0.009	0.002	0.001	876.51	570
0.001	0.012	0.013	0.016	0.009	0.002	0.001	871.45	570
0.001	0.01	0.013	0.01	0.011	0.001	0.001	854.82	570
0.002	0.01	0.016	0.018	0.012	0.002	0.002	886.67	570
0.001	0.01	0.012	0.013	0.009	0.002	0.001	872.09	570
0.001	0.01	0.012	0.013	0.009	0.002	0.001	887.72	570
0.001	0.01	0.012	0.013	0.009	0.002	0.001	878.58	570
0.001	0.01	0.012	0.013	0.009	0.002	0.001	885.72	570
0.001	0.01	0.012	0.013	0.009	0.002	0.001	886.43	570
0.001	0.01	0.012	0.015	0.008	0	0.002	890.52	570
0.001	0.01	0.012	0.015	0.008	0	0.002	887.83	570
0.001	0.01	0.012	0.015	0.008	0	0.002	890.28	570
0.001	0.01	0.012	0.015	0.008	0	0.002	862.87	570
0.001	0.01	0.012	0.015	0.008	0	0.002	888.37	570
0.002	0.01	0.016	0.018	0.012	0.002	0.002	897.84	570
0.001	0.009	0.011	0.015	0.01	0	0.001	873.51	570
0.001	0.014	0.013	0.015	0.007	0.001	0.001	887.33	570
0.001	0.01	0.016	0.01	0.013	0.001	0.001	876.67	570
0.001	0.01	0.016	0.01	0.013	0.001	0.001	886.7	570
0.001	0.01	0.016	0.01	0.013	0.001	0.001	879.47	570
0.001	0.01	0.013	0.016	0.012	0.001	0.001	884.6	570
0.001	0.01	0.016	0.01	0.013	0.001	0.001	879.02	570

YS	UTS	EL
263	357	40
242	363	38
318	367	37
285	367	39
283	355	43
306	369	40
283	383	40
302	356	42
291	367	42
286	367	42
288	365	41
265	357	43
265	357	43
278	365	43
285	359	42
285	359	42
285	359	42
283	366	42
283	366	42
288	365	41
304	372	43
304	372	43
307	359	39
283	357	41
292	368	40
262	333	45
298	383	40
295	370	42
285	365	41
305	372	37
315	378	37
315	379	36
305	375	38
305	375	38
308	372	37
295	372	40
315	378	38
315	378	38
318	379	37
311	378	38
294	354	40
282	356	43
296	358	40
278	369	43
294	367	42
293	369	43
325	385	45
324	387	42
311	372	37

317	368	43
284	364	43
315	368	42
296	368	38
274	353	41
305	369	39
305	368	41
298	376	38
287	363	37
338	384	35
274	352	40
282	347	39
282	347	39
309	355	38
301	349	38
298	347	39
298	347	39
298	347	39
292	356	38
294	355	38
313	370	38
303	361	38
313	370	38
310	374	38
301	370	38
302	371	40
300	364	40
302	371	40
302	371	40
300	368	38
284	367	38
284	367	38
286	365	38
288	368	40
279	361	40
288	368	40
281	337	40
272	332	40
281	337	40
288	368	38
311	382	38
288	366	43
291	378	42
281	357	44
291	378	42
291	378	42
281	357	44
286	361	42
286	361	42
288	368	40

276	359	44
276	362	42
267	353	42
254	342	40
254	342	40
267	353	42
272	361	42
272	361	42
284	366	43
291	367	42
291	367	42
258	348	43
281	362	45
275	362	45
292	362	41
310	373	40
310	373	40
313	372	41
283	357	39
295	368	38
291	353	38
296	367	38
323	379	37
323	379	37
301	369	37
301	369	37
301	369	37
317	359	36
323	371	35
323	371	35
325	376	36
292	366	44
338	392	34
338	392	34
328	385	36
328	385	36
318	372	40
309	363	38
300	361	38
309	363	38
287	365	40
286	354	40
316	369	38
305	372	37
289	348	38
305	372	37
301	379	38
306	370	38
306	370	38
277	360	38

299	377	37
275	355	38
282	365	40
282	365	40
337	397	38
317	369	40
294	367	42
294	367	42
287	371	42
274	339	42
288	369	40
296	352	42
296	352	42
305	364	42
305	364	42
298	363	42
305	372	37
297	365	40
294	364	40
294	364	40
298	363	42
287	355	40
287	355	40
316	373	40
291	367	40
291	367	40
297	365	40
295	379	40
287	382	41
305	376	39
296	374	39
308	378	38
308	378	38
306	372	37
313	368	37
315	376	38
312	378	38
311	369	38
285	367	41
266	358	38
268	353	38
268	353	38
278	357	38
278	357	38
269	348	40
268	338	40
268	338	40
269	342	40
271	344	40
269	342	40

271	344	40
276	349	40
269	342	40
268	344	40
268	344	40
283	361	38
283	371	38
287	369	40
287	369	40
250	322	42
242	319	44
251	324	44
248	323	42
251	324	44
253	325	44
291	371	38
295	376	39
275	350	40
275	350	40
273	353	40
279	369	38
293	369	42
291	368	40
291	368	40
289	369	40
289	369	40
292	351	42
292	351	42
316	375	42
313	377	40
316	375	42
313	377	40
313	377	40
313	377	40
302	355	40
313	377	40
282	352	38
298	379	42
310	375	40
310	375	40
293	360	42
310	375	40
310	375	40
310	375	40
310	375	40
299	374	42
310	375	40
293	360	42
294	369	42
293	360	42

293	360	42
293	360	42
278	365	41
269	358	40
269	358	40
285	367	41
285	367	41
278	365	41
321	390	37
316	388	38
315	382	38
315	382	38
318	382	37
315	389	37
306	378	37
315	378	38
315	378	38
315	378	38
315	378	38
315	378	38
299	371	37
305	376	38
305	376	37
315	380	38
305	376	38
310	378	40
308	381	41
311	378	37
315	380	38
298	378	38
307	376	38
307	376	38
307	376	38
298	378	38
298	378	38
305	389	40
291	353	38
298	375	37
299	352	38
298	375	37
298	375	37
298	375	37
288	352	40
287	354	38
296	370	38
305	384	37
305	377	38
305	384	37
305	377	38
303	378	38

305	377	38
311	374	38
311	374	38
261	341	40
261	341	40
261	341	40
278	344	40
272	339	40
290	373	38
303	385	40
299	367	39
296	346	40
299	376	40
299	376	40
288	359	38
288	359	38
317	369	38
317	369	38
254	331	44
249	341	42
254	331	44
262	329	44
249	317	44
258	328	42
248	334	44
293	354	43
304	377	43
294	351	43
293	354	43
321	393	40
321	393	40
328	389	40
321	393	40
328	389	40
312	374	43
318	394	41
318	394	41
271	364	45
280	359	46
280	359	46
282	360	45
280	362	42
315	358	45
298	376	42
298	376	42
289	361	44
308	369	42
289	361	44
289	361	44
292	376	42

292	376	42
291	362	44
284	365	44
284	365	44
284	365	44
283	362	44
283	362	44
283	362	44
321	376	43
321	376	43
281	357	42
272	334	43
272	334	43
277	359	45
283	359	45
283	359	45
272	359	44
279	359	43
303	367	43
276	362	43
284	357	44
284	357	44
315	353	40
292	357	38
315	353	40
292	357	38
292	357	38
299	353	38
291	368	38
291	368	38
282	353	40
278	361	42
288	364	37
287	371	38
291	366	38
335	387	38
306	387	38
303	377	38
303	377	38
311	378	38
311	378	38
291	360	41
298	367	41
276	358	37
285	366	38
267	360	42
267	360	42
275	362	41
275	362	41
269	360	41

269	360	41
278	365	41
307	372	39
298	375	39
278	365	41
298	375	39
290	353	41
290	353	41
295	371	41
301	372	41
282	362	38
301	372	41
288	367	38
282	362	38
286	369	38
286	369	38
301	357	36
311	381	35
303	381	34
303	381	34
306	378	36
306	378	36
306	378	36
302	379	35
302	379	35
308	378	36
308	378	36
308	378	36
276	362	41
296	371	40
280	365	40
320	379	40
277	363	40
277	363	40
277	363	40
321	381	40
